# Supplementary figures and images for: Cohort profile: the Pregnancy Risk Infant Surveillance and Measurement Alliance (PRISMA) – Pakistan
Source: BMJ Open. 2023 Dec 9;13(12):e078222. doi: 10.1136/bmjopen-2023-078222 (PMC10729021; doi:10.1136/bmjopen-2023-078222)

Supplemental Figure 1. PRISMA study sites in Karachi, Pakistan

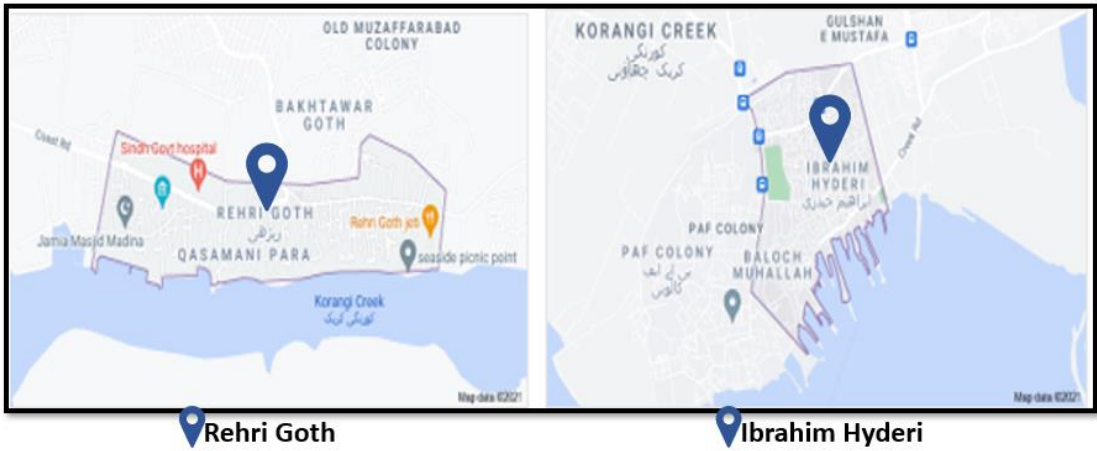

Supplement: Supplementary data [file bmjopen-2023-078222supp001.pdf]
