# Supplementary material for: Cohort profile: the Pregnancy Risk Infant Surveillance and Measurement Alliance (PRISMA) – Pakistan
Source: BMJ Open. 2023 Dec 9;13(12):e078222. doi: 10.1136/bmjopen-2023-078222 (PMC10729021; doi:10.1136/bmjopen-2023-078222)
Supplement: Author's manuscript [file bmjopen-2023-078222.draft_revisions.pdf]

BMJ Open

BMJ Open is committed to open peer review. As part of this commitment we make the peer review history of every article we publish publicly available.

When an article is published we post the peer reviewers' comments and the authors' responses online. We also post the versions of the paper that were used during peer review. These are the versions that the peer review comments apply to.

The versions of the paper that follow are the versions that were submitted during the peer review process. They are not the versions of record or the final published versions. They should not be cited or distributed as the published version of this manuscript.

BMJ Open is an open access journal and the full, final, typeset and author-corrected version of record of the manuscript is available on our site with no access controls, subscription charges or pay-per-view fees (http://bmjopen.bmj.com).

If you have any questions on BMJ Open's open peer review process please email info.bmjopen@bmj.com

BMJ Open

Cohort profile: The Pregnancy Risk Infant Surveillance and Measurement Alliance (PRISMA) - Pakistan

Journal:	*BMJ Open*
Manuscript ID	bmjopen-2023-078222
Article Type:	Cohort profile
Date Submitted by the Author:	27-Jul-2023

Complete List of Authors:	Naz, Sabahat; The Aga Khan University, Department of Pediatrics and Child Health<br>Jaffar, Ali; The Aga Khan University, Department of Pediatrics and Child Health<br>Kashif, Muhammad ; The Aga Khan University, Department of Pediatrics and Child Health<br>Hussain, Zaid; The Aga Khan University, Department of Pediatrics and Child Health<br>Yazdani, Nida; VITAL Pakistan<br>Khan, Uzma; VITAL Pakistan<br>Farooq, Fouzia; The George Washington University, Department of Global Health<br>Nisar, Muhammad; The Aga Khan University, Department of Pediatrics and Child Health<br>Jehan, Fyezah; The Aga Khan University, Department of Pediatrics and Child Health<br>Smith, Emily ; The George Washington University, Public Health<br>Hoodbhoy, Zahra; The Aga Khan University, Department of Pediatrics and Child Health

Keywords:	Follow-Up Studies, Community child health < PAEDIATRICS, Primary Health Care, PUBLIC HEALTH, Risk Factors

SCHOLARONE™
Manuscripts

I, the Submitting Author has the right to grant and does grant on behalf of all authors of the Work (as defined in the below author licence), an exclusive licence and/or a non-exclusive licence for contributions from authors who are: i) UK Crown employees; ii) where BMJ has agreed a CC-BY licence shall apply, and/or iii) in accordance with the terms applicable for US Federal Government officers or employees acting as part of their official duties; on a worldwide, perpetual, irrevocable, royalty-free basis to BMJ Publishing Group Ltd ("BMJ") its licensees and where the relevant Journal is co-owned by BMJ to the co-owners of the Journal, to publish the Work in this journal and any other BMJ products and to exploit all rights, as set out in our licence.

The Submitting Author accepts and understands that any supply made under these terms is made by BMJ to the Submitting Author unless you are acting as an employee on behalf of your employer or a postgraduate student of an affiliated institution which is paying any applicable article publishing charge ("APC") for Open Access articles. Where the Submitting Author wishes to make the Work available on an Open Access basis (and intends to pay the relevant APC), the terms of reuse of such Open Access shall be governed by a Creative Commons licence – details of these licences and which Creative Commons licence will apply to this Work are set out in our licence referred to above.

Other than as permitted in any relevant BMJ Author's Self Archiving Policies, I confirm this Work has not been accepted for publication elsewhere, is not being considered for publication elsewhere and does not duplicate material already published. I confirm all authors consent to publication of this Work and authorise the granting of this licence.

For peer review only

Title: Cohort profile: The Pregnancy Risk Infant Surveillance and Measurement Alliance (PRISMA) Pakistan

Authors: Sabahat Naz[1], Ali Jaffar[1], Muhammad Kashif[1], Zaid Hussain[1], Nida Yazdani[2], Uzma Khan[2], Fouzia Farooq[3], Imran Nisar[1], Fyezah Jehan[1], Emily R. Smith[3], Zahra Hoodbhoy[1]

[1]Department of Pediatrics and Child Health, The Aga Khan University, Stadium Road, Karachi, Pakistan 74800

[2]VITAL Pakistan Trust, Karachi, Pakistan

[3]Department of Global Health, Milken Institute School of Public Health, The George Washington University, Washington, D.C. 20052, USA

Corresponding author:

Zahra Hoodbhoy

Department of Pediatrics and Child Health

The Aga Khan University

Stadium Road, Karachi, Pakistan 74800

Tel No: 92-2134864126

Email: zahra.hoodbhoy@aku.edu

For peer review only

Abstract

Purpose:

Pakistan has disproportionately high maternal and neonatal morbidity and mortality. There is a lack of accurate population-level data to provide evidence for risk factors, morbidities, and mortality among pregnant women and their newborns. Pregnancy Risk Infant Surveillance and Measurement Alliance (PRISMA) is a multi-country effort that aims to collect high-dimensional, standardized data across five South Asian and African countries to estimate risk and develop innovative strategies to optimize pregnancy outcomes. This study presents the baseline maternal and neonatal characteristics of the Pakistan site.

Participants:

PRISMA Pakistan study is being conducted at two peri-urban field sites in Karachi, Pakistan. These sites have primary health care clinics where pregnant women and their newborns are followed during the antenatal, intrapartum, and postnatal periods up to one year after delivery. All encounters are captured electronically through a custom-built android application. A total of 3,731 pregnant women with a mean age of 26.6 ± 5.8 years at the time of pregnancy with neonatal outcomes between January 2021 to August 2022 serve as a baseline for the PRISMA Pakistan study.

Findings to date:

In this cohort, live births accounted for the majority of pregnancy outcomes (92%, n=3,478), followed by miscarriages/abortions (5.5%, n=205) and stillbirths (2.6%, n=98). Twenty-two percent of women (n=786) delivered at home. One out of every four neonates was low birth weight (< 2500 grams), and one out of every five was preterm (gestational age <37 weeks). The maternal mortality rate was 172/100,000 pregnancies, the neonatal mortality rate was 52/1000 live births, and the stillbirth rate was 27/1000 births.

For peer review only - http://bmjopen.bmj.com/site/about/guidelines.xhtml

The three most common causes of neonatal deaths reported were perinatal asphyxia (39.6%), preterm births (19.8%), and infections (12.6%).

Future plans:

The PRISMA cohort will provide data-driven insights to prioritize and design interventions to improve maternal and neonatal outcomes in low-resource regions.

For peer review only

For peer review only - http://bmjopen.bmj.com/site/about/guidelines.xhtml

Strengths and limitations of this study

- This is a unique longitudinal cohort in Pakistan with the ability to leverage an existing demographic surveillance system to capture early pregnancies and follow these women postpartum with low attrition rates.
- Since this cohort exists in a peri-urban setting, the population included is representative of such settings in low-resource countries.
- This cohort is collecting harmonized data obtained using standardized data collection forms across other similar LMICs, enabling further cross-country comparison for MNCH outcomes.
- Although women are encouraged to deliver at the recommended referral facility, nearly 40% may deliver at other health facilities or their homes. In such cases, self-report is being used to assess key labor and delivery outcomes, thus limiting its interpretation.

For peer review only

For peer review only - http://bmjopen.bmj.com/site/about/guidelines.xhtml

INTRODUCTION:

Population-based longitudinal birth cohorts provide important data to study a life course approach to in-utero exposures and etiological factors that contribute to developing diseases early and later in life (1). The advantages of such cohort studies include standardized data collection, a systematic approach in following participants throughout the study, and the generalizability of findings to the larger population (1). In addition, it allows investigators to collect accurate information on the exposures, outcomes, potential confounders, and other important characteristics at the individual as well as population level (2). Early identification of these risk factors may provide an opportunity to design and implement population-based preventive strategies to optimize individual and population-level outcomes (3).

However, there is a dearth of such longitudinal data from low-and middle-income countries (LMICs), particularly related to well-measured risk factors, well-defined morbidities, and mortality outcomes among pregnant women and their newborns (4). The availability of smaller datasets in these geographic regions limits the field of Maternal and Newborn Health (MNH) to efficiently develop timely interventions to prevent or manage poor maternal and neonatal outcomes (5). Further, population-based longitudinal studies that provide robust data on pregnancy risk factors such as sociodemographic, clinical features, antenatal (ANC), and postnatal care (PNC) can aid in the development of stratified care pathways for efficient resource utilization and improve outcomes (3, 6).

To address these gaps, the Pregnancy Risk Infant Surveillance and Measurement Alliance (PRISMA) study is being conducted across five sites in South Asia (India, Pakistan) and Sub-Saharan Africa (Zambia, Kenya, Ghana) to assess pregnancy risk factors and their relationships with poor pregnancy outcomes, such as stillbirth, neonatal morbidity and mortality, and maternal mortality and severe morbidity. The objective of this open, multi-country prospective cohort study is to provide a consistent data set to quantify the burden of maternal, fetal, and neonatal risk factors and their associated outcomes in LMICs and to better understand vulnerabilities, morbidity, and death. Each country had a pre-harmonization baseline period to assess burden of adverse maternal and neonatal outcomes in the study area. PRISMA data would play a pivotal role in the development of innovative preventive measures to optimize pregnancy outcomes for mothers and their newborns.

For peer review only - http://bmjopen.bmj.com/site/about/guidelines.xhtml

COHORT DESCRIPTION:

Study design and participants

In Pakistan, PRISMA study is being conducted in two peri-urban community settings in Karachi called Rehri Goth and Ibrahim Hyderi; the areas comprise approximately 23,170 households and a population of 227,549 individuals (refer to Supplemental Figure 1).

The existing maternal and child health surveillance system at these sites is being leveraged for the early identification of pregnancies. Pregnant women captured through pregnancy surveillance are encouraged to visit the primary health care (PHC) clinics located within the community for antenatal and postnatal care. Pregnant women aged 15 - 49 years with a confirmed intrauterine pregnancy of <20 weeks via ultrasound are eligible to participate in the study. Women with ectopic or molar pregnancies or those who plan to move outside the study catchment area during pregnancy or 12 months after delivery are excluded. A total of 3,731 pregnant women with a neonatal outcome between January 2021 to August 2022 serve as a baseline for PRISMA Pakistan. The enrollment flow chart and baseline characteristics of this population are described in Figure 1 and Table 1, respectively.

Figure 1. Flow diagram for PRISMA enrollment status January 2021 to August 2022

For peer review only

For peer review only - http://bmjopen.bmj.com/site/about/guidelines.xhtml

Table 1. Baseline Characteristics of PRISMA study participants

Characteristics	Total Population (n = 3,731)<br>Mean ± SD, Median (range) or n (%)
Age at marriage (years)	20.1 ± 3.2
**Maternal education**	
Literate	1,198 (33.1)
**Women's employment status**	
Employed	1,129 (31.2)
**Husband's employment status**	
Employed	3,582 (98.9)
**Wealth Index (quintiles)**	
Q1 (least wealthy)	791 (21.8)
Q2	659 (18.2)
Q3	736 (20.3)
Q4	813 (22.4)
Q5 (wealthiest)	624 (17.2)
Reported Smoking	95 (2.6)
Current use of smokeless tobacco	818 (21.9)
Total number of pregnancies	3 (1 - 16)
Primigravida	836 (22.4)
Multigravida (2 – 5)	2,232 (59.8)
Grand Multigravida (>5)	663 (17.8)
No. of previous livebirths	2 (1 - 14)
No. of previous stillbirths	1 (1 - 5)
No. of previous miscarriages	1 (1 - 10)
No. of previous induced abortions	1 (1 - 3)

Measures and procedure

PRISMA study visits include five antenatal visits as follows: enrollment (gestational age (GA) <20 weeks), 20 weeks, 28 weeks, 32 weeks, and 36 weeks of gestation. All ANC visits are conducted at the PHC located within the

For peer review only - http://bmjopen.bmj.com/site/about/guidelines.xhtml

catchment area. Data is also collected at the time of delivery (at the facility or home) as well as at six PNC visits (i.e., 3 days, 1 week, 4 weeks, 6 weeks, 26 weeks, and 52 weeks postpartum) for both mothers and infants. All PNC visits are conducted at home except for the 6-week PNC visit that occurs at the PHC to comply with the child's immunization schedule.

Data for this study is being collected prospectively by interviewing study participants as well as retrieving relevant information from existing clinical records. The information at each antenatal visit includes medical and obstetric history, pregnancy status, current clinical status, medications and treatment during pregnancy, hospitalizations, and health promotion activities, while information regarding labor and delivery, maternal and newborn complications, and infant growth are recorded during the postnatal visits. Table 2 summarizes the details regarding the information collected in the study.

Table 2. Summary of data collection throughout the study

**Study variables**	**Enrollment** <20 weeks	**ANC 20** 18-22 weeks	**ANC 28** 26-30 weeks	**ANC 32** 31-33 weeks	**ANC 36** 34 – 40 weeks	**IPC** Delivery – 72 hours	**PNC 0** 3-5 days	**PNC 1** 7-14 days	**PNC 42** 28-35 days	**PNC 6** 6-7 weeks	**PNC 26** 26-28 weeks	**PNC 52** 52-54 weeks
Sociodemographic information	X											
Pregnancy history	X											
Ultrasound	X			X								
Maternal anthropometrics	X	X	X	X	X	X	X	X	X	X	X	X
ANC clinical status		X	X	X	X							
Diagnostics		X	X	X	X	X	X	X	X	X		
IPC clinical status						X						
Delivery outcomes						X	X					
Newborn anthropometrics							X	X	X	X	X	X
PNC clinical status							X	X	X	X	X	X

ANC= Antenatal care, IPC= Intrapartum care, PNC= Postnatal care

For peer review only

All visits are captured electronically through a custom build android application. This application was built on Open Smart Register Platform, an open-source mobile health platform designed to support the WHO SMART guidelines for data-driven decision-making in healthcare (7). Once data is entered into the application, it is extracted, transformed, and loaded into a PostgreSQL (8) relational database management system to allow for monitoring, reporting, and analytics. The application is user-specific and team-based (midwives, postnatal visits team, etc.), with each team having a unique ID and password for data collection.

Patient and public involvement:

PRISMA cohort participants were not involved in the design or recruitment stage of the study. However, the community mobilization teams of PRISMA have been actively engaged with community stakeholders and pregnant women in the community to inform them regarding the study and benefits of early initiation of antenatal care. Community mobilization is an ongoing exercise for the PRISMA Pakistan study.

FINDINGS TO DATE:

As of August 2022, 3,731 women with pregnancy outcomes serve as a baseline for the PRISMA Pakistan cohort (Table 3). Women enrolled in this cohort have a mean age of 26.6 ± 5.8 years at the time of pregnancy. The average number of antenatal visits were 5.4 ± 2.8, with a mean GA of 17.2 ± 8.1 weeks at the first visit. Using the cutoff of mid-upper arm circumference (MUAC) of <23 cm, 27% (n=1,006) of pregnant were malnourished. Approximately two-thirds of women (64%, n=2570) had either mild, moderate, or severe anemia. Majority of the pregnancy outcomes in this cohort were live births (92%, n=3,478) followed by miscarriages/abortions (5.5%, n=205) and stillbirths (2.6%, n=98). Twenty-two percent (n=786) of these women delivered at home. Overall, 20.1% (n=696) infants were preterm, and 24.1% (n=611) were low birthweight. Detailed pregnancy and delivery characteristics and neonatal outcomes are described in Table 3.

For peer review only

For peer review only - http://bmjopen.bmj.com/site/about/guidelines.xhtml

Table 3. Pregnancy and delivery characteristics and neonatal outcomes in the study population

Pregnancy Characteristics	Total Population (n = 3,731) Mean +/- SD; n (%)
Age at the time of pregnancy (years)	26.6 ± 5.8
Number of ANC visits	5.4 ± 2.8
GA at first ANC visit (weeks)	17.2 ± 8.1
MUAC* (cm)	25.8 ± 4.1
**Nutritional status based on MUAC (cm)**	
Normal (≥23)	2,724 (73.0)
Malnourished (<23)	1,006 (27.0)
BMI* (kg/m^2)	23.4 ± 5.2
**Nutritional status based on BMI (kg/m^2)**	
Underweight (<18.5)	564 (15.1)
Normal weight (18.5 – 22.9)	1,454 (39.0)
Overweight (23.0 – <26.9)	891 (23.9)
Obese (≥27.0)	821 (22.0)
Hemoglobin levels* (g/dL)	10.3 ± 1.4
**Anemia status**	
Normal (≥11.0)	1,161 (35.7)
Mild Anemia (10.0 - 10.9)	940 (28.9)
Moderate Anemia (7.0 - 9.9)	1,077 (33.1)
Severe Anemia (<7.0)	71 (2.2)
Chronic Hypertension	124 (3.3)
Diabetes Mellitus	23 (0.6)
Gestational Hypertension	48 (1.3)
Hepatitis B	33 (1.2)
Hepatitis C	52 (1.9)
**Delivery Characteristics and Neonatal outcomes**	**Total Population (n = 3,576) n (%)**
**Number of fetuses**	
Single	3,618 (98.7)
Twins	48 (1.3)

For peer review only - http://bmjopen.bmj.com/site/about/guidelines.xhtml

**Birth outcomes**	
Livebirths	3,478 (92.0)
Stillbirths	98 (2.6)
Miscarriages	180 (4.8)
Abortions	25 (0.7)
**Gender**	
Male	1,836 (51.3)
Female	1,738 (48.6)
**Place of delivery**	
Facility-based	2,790 (78.0)
Home births	786 (22.0)
**Mode of delivery**	
Vaginal	2,620 (73.3)
Cesarean	956 (26.7)
Elective	530 (55.7)
Emergent	422 (44.3)
**Gestational age at delivery (in weeks)**	
Extremely Preterm (<28)	8 (0.2)
Very Preterm (28 - <32)	44 (1.3)
Moderate to Late Preterm (32 - < 37 weeks)	644 (18.6)
Term (≥37)	2,761 (79.9)
**Birth weight categories (in kg)**	
Extremely Low Birth weight (<1)	2 (0.1)
Very Low Birth weight (1 – < 1.5)	31 (1.2)
Low Birth Weight (1.5 - <2.5)	578 (22.8)
Normal Birth Weight (≥2.5)	1,930 (75.9)

BMI, Body mass index; GA, Gestational age; MUAC, Mid-upper arm circumference

There were 98 stillbirths in the PRiSMA cohort, with 75% intrapartum stillbirths (n=74) and 25% antepartum stillbirths (n=24). Nearly three-quarters of neonatal deaths (76.4%, n=139) occurred during the first week of life. The three main causes of death during the neonatal period were perinatal asphyxia (39.6%, n=72), preterm birth (19.8%, n=36), and infections (12.6%, n=23).

Maternal and neonatal mortality rates are described in Table 4. In the PRISMA cohort, the maternal mortality rate is 172 per 100,000 live births, which is lower than the national maternal mortality (186 per 100,000 live births) (9). Rate of stillbirths in this cohort is 27 per 1000 births, which is also lower than the national rate of 44 per 1,000 live births (10). On the other hand, the neonatal mortality rates in the PRISMA cohort (52 per 1,000 live births) are comparable with the national neonatal mortality rates (51 per 1,000 livebirths) (10). The lower rates of maternal

For peer review only - http://bmjopen.bmj.com/site/about/guidelines.xhtml

mortality and stillbirth observed in this cohort may be attributed to the provisions of adequate antenatal care as well as timely referrals when needed. However, postnatal care primarily takes place at home, and the lack of follow-up could potentially explain the higher neonatal mortality.

Table 4. Rates of maternal and neonatal mortality and stillbirth in PRISMA Pakistan

Indicators	Mortality rates in PRISMA cohort	Mortality rates in Pakistan (2018-19) (9, 10)
**Maternal mortality rate (MMR)**	172 / 100,000 livebirths	186 / 100,000 livebirths
**Stillbirth rate**	27 Stillbirths / 1,000 births	44 Stillbirths / 1,000 births
**Neonatal mortality rate (NMR)**	52 / 1,000 livebirths	51 / 1,000 livebirths

STRENGTHS AND LIMITATIONS OF THIS STUDY

A key strength of the PRiSMA Pakistan cohort is its ability to leverage the existing demographic surveillance system to capture pregnancies early in the first trimester and follow these women for up to one year after delivery. Since this cohort leverages the community-based setting, the population included in this cohort is nationally representative of its sample. Another major strength of this cohort is the collection of harmonized data obtained using standardized CRFs across four other LMICs, enabling further cross-country comparison for maternal, newborn, and child health outcomes. One limitation of this study is that although women are encouraged to deliver at the recommended referral facility, nearly 40% may deliver outside the health facility, including their homes. In such cases, self-report is being used to assess key labor and delivery outcomes, thus limiting its interpretation. Another limitation is that not all women from the community are seeking care during pregnancy, and hence may be different from those enrolled in PRISMA, thus limiting the generalizability. However, with the existing baseline data acquired through the demographic surveillance system, the study team will be able to compare any differences in health-seeking behaviors in order to make the study findings more generalizable.

For peer review only - http://bmjopen.bmj.com/site/about/guidelines.xhtml

COLLABORATION

The study protocol for the harmonized ongoing PRISMA cohort is registered with https://clinicaltrials.gov (number: NCT05656352) or by contacting the corresponding author of this manuscript.

Acknowledgments: The authors acknowledge the contributions of all women who participated in the PRISMA study and the PRISMA study team.

Contributors:

ZH, FJ, IN, FF, and ES conceptualized the study and secure funding. ZH, NY, and UK supervised the study at field sites. ZH, SN, ZHU, AJ, NY, and UK were involved in the analysis, interpretation, and writing manuscript. ZH, FJ, IN, FF, and ES reviewed and provided scientific revision to the manuscript. All authors agreed prior to submission to take responsibility and be accountable for the contents of the manuscript. All authors read and approved the final manuscript.

Funding Statement: This study is funded by the Bill & Melinda Gates Foundation (INV-005776**).**

Competing Interests: None declared

Ethics statement: The PRISMA MNH study was approved by the George Washington University's Committee on Human Research (IRB: FWA00005945) and the Ethical Review Committee of the Vital Pakistan Trust (001-VPT-IRB-20).

For peer review only

For peer review only - http://bmjopen.bmj.com/site/about/guidelines.xhtml

References:

1. Manolio TA, Bailey-Wilson JE, Collins FS. Genes, environment and the value of prospective cohort studies. Nat Rev Genet. 2006;7(10):812-20.
2. Lawlor DA, Andersen AM, Batty GD. Birth cohort studies: past, present and future. Int J Epidemiol. 2009;38(4):897-902.
3. Baqui A, Ahmed P, Dasgupta SK, Begum N, Rahman M, Islam N, et al. Development and validation of a simplified algorithm for neonatal gestational age assessment - protocol for the Alliance for Maternal Newborn Health Improvement (AMANHI) prospective cohort study. J Glob Health. 2017;7(2):021201.
4. Williams E. Are low- and middle-income countries collecting priority maternal and newborn health service data? March 28, 2019.
5. Kitila SB, Feyissa GT, Olika AK, Wordofa MA. Maternal Healthcare in Low- and Middle-Income Countries: A Scoping Review. Health Services Insights. 2022;15:11786329221100310.
6. Goldenberg RL, Saleem S, Ali S, Moore JL, Lokangako A, Tshefu A, et al. Maternal near miss in low-resource areas. Int J Gynaecol Obstet. 2017;138(3):347-55.
7. [Available from: https://smartregister.org/#:~:text=OpenSRP%20is%20a%20%E2%80%9Cnext%20gen,systems%20like%20DHIS2%20or%20OpenMRS.
8. Momjian B. PostgreSQL Introduction and Concepts Bruce Momjian ADDISON. 2001.
9. National Institute of Population Studies (NIPS) [Pakistan] and ICF. 2020. Pakistan Maternal Mortality Survey 2019: Key Indicators Report. Islamabad, Pakistan, and Rockville, Maryland, USA: NIPS and ICF.
10. Aziz A, Saleem S, Nolen TL, Pradhan NA, McClure EM, Jessani S, et al. Why are the Pakistani maternal, fetal and newborn outcomes so poor compared to other low and middle-income countries? Reprod Health. 2020;17(Suppl 3):190.

For peer review only - http://bmjopen.bmj.com/site/about/guidelines.xhtml

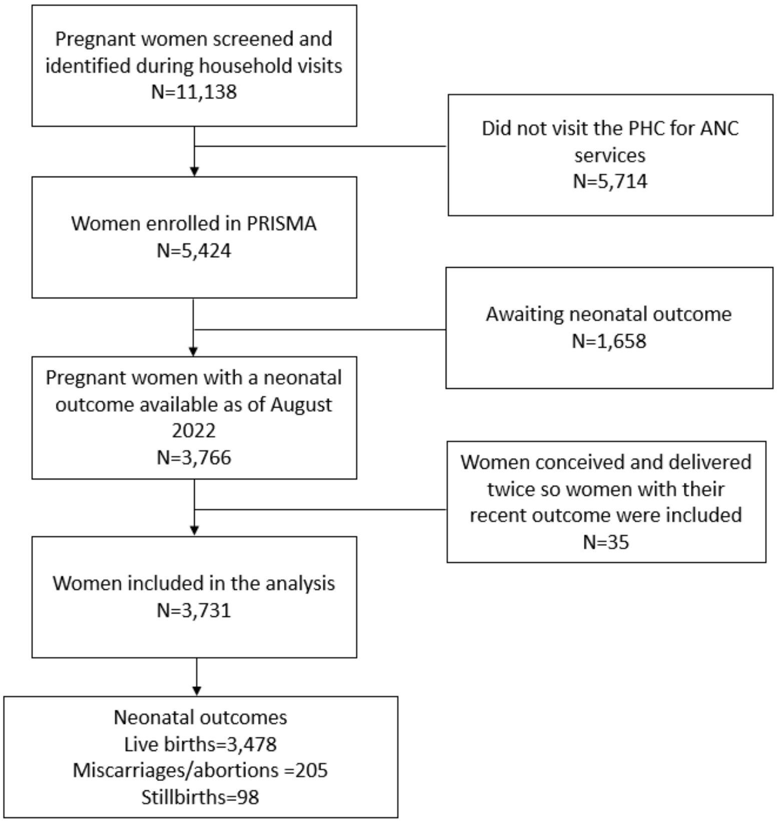

PHC= Primary healthcare, ANC= Antenatal care

Supplemental Figure 1. PRISMA study sites in Karachi, Pakistan

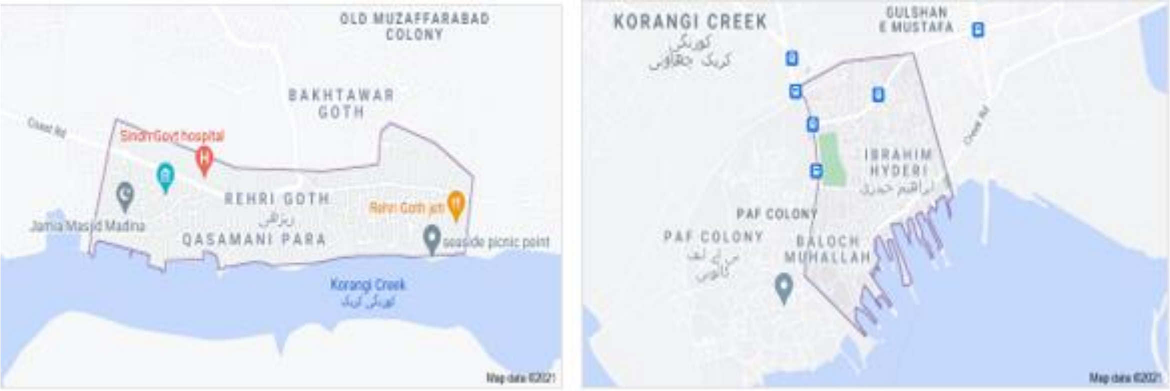

Rehri Goth **Ibrahim Hyderi**

For peer review only

STROBE Statement—Checklist of items that should be included in reports of ***cohort studies***

	Item No	Recommendation	Page No
**Title and abstract**	1	(*a*) Indicate the study's design with a commonly used term in the title or the abstract	1
		(*b*) Provide in the abstract an informative and balanced summary of what was done and what was found	2-3
**Introduction**			
Background/rationale	2	Explain the scientific background and rationale for the investigation being reported	5
Objectives	3	State specific objectives, including any prespecified hypotheses	5
**Methods**			
Study design	4	Present key elements of study design early in the paper	6
Setting	5	Describe the setting, locations, and relevant dates, including periods of recruitment, exposure, follow-up, and data collection	6
Participants	6	(*a*) Give the eligibility criteria, and the sources and methods of selection of participants. Describe methods of follow-up	6
		(*b*) For matched studies, give matching criteria and number of exposed and unexposed	NA
Variables	7	Clearly define all outcomes, exposures, predictors, potential confounders, and effect modifiers. Give diagnostic criteria, if applicable	NA
Data sources/ measurement	8*	For each variable of interest, give sources of data and details of methods of assessment (measurement). Describe comparability of assessment methods if there is more than one group	7-9
Bias	9	Describe any efforts to address potential sources of bias	NA
Study size	10	Explain how the study size was arrived at	NA
Quantitative variables	11	Explain how quantitative variables were handled in the analyses. If applicable, describe which groupings were chosen and why	NA
Statistical methods	12	(*a*) Describe all statistical methods, including those used to control for confounding	NA
		(*b*) Describe any methods used to examine subgroups and interactions	NA
		(*c*) Explain how missing data were addressed	NA
		(*d*) If applicable, explain how loss to follow-up was addressed	NA
		(*e*) Describe any sensitivity analyses	NA
**Results**			
Participants	13*	(a) Report numbers of individuals at each stage of study—eg numbers potentially eligible, examined for eligibility, confirmed eligible, included in the study, completing follow-up, and analysed	6
		(b) Give reasons for non-participation at each stage	NA
		(c) Consider use of a flow diagram	6
Descriptive data	14*	(a) Give characteristics of study participants (eg demographic, clinical, social) and information on exposures and potential confounders	7
		(b) Indicate number of participants with missing data for each variable of interest	NA
		(c) Summarise follow-up time (eg, average and total amount)	8-9
Outcome data	15*	Report numbers of outcome events or summary measures over time	NA

For peer review only - http://bmjopen.bmj.com/site/about/guidelines.xhtml

Main results	16	(*a*) Give unadjusted estimates and, if applicable, confounder-adjusted estimates and their precision (eg, 95% confidence interval). Make clear which confounders were adjusted for and why they were included	NA
		(*b*) Report category boundaries when continuous variables were categorized	NA
		(*c*) If relevant, consider translating estimates of relative risk into absolute risk for a meaningful time period	NA
Other analyses	17	Report other analyses done—eg analyses of subgroups and interactions, and sensitivity analyses	NA
**Discussion**			
Key results	18	Summarise key results with reference to study objectives	9-12
Limitations	19	Discuss limitations of the study, taking into account sources of potential bias or imprecision. Discuss both direction and magnitude of any potential bias	12-13
Interpretation	20	Give a cautious overall interpretation of results considering objectives, limitations, multiplicity of analyses, results from similar studies, and other relevant evidence	9-12
Generalisability	21	Discuss the generalisability (external validity) of the study results	12
**Other information**			
Funding	22	Give the source of funding and the role of the funders for the present study and, if applicable, for the original study on which the present article is based	13

*Give information separately for exposed and unexposed groups.

Note: An Explanation and Elaboration article discusses each checklist item and gives methodological background and published examples of transparent reporting. The STROBE checklist is best used in conjunction with this article (freely available on the Web sites of PLoS Medicine at http://www.plosmedicine.org/, Annals of Internal Medicine at http://www.annals.org/, and Epidemiology at http://www.epidem.com/). Information on the STROBE Initiative is available at http://www.strobe-statement.org.

For peer review only

For peer review only - http://bmjopen.bmj.com/site/about/guidelines.xhtml

BMJ Open

Cohort profile: The Pregnancy Risk Infant Surveillance and Measurement Alliance (PRISMA) - Pakistan

Journal:	*BMJ Open*
Manuscript ID	bmjopen-2023-078222.R1
Article Type:	Cohort profile
Date Submitted by the Author:	16-Oct-2023
Complete List of Authors:	Naz, Sabahat; The Aga Khan University, Department of Pediatrics and Child Health<br>Jaffar, Ali; The Aga Khan University, Department of Pediatrics and Child Health<br>Yazdani, Nida; VITAL Pakistan<br>Kashif, Muhammad ; The Aga Khan University, Department of Pediatrics and Child Health<br>Hussain, Zaid; The Aga Khan University, Department of Pediatrics and Child Health<br>Khan, Uzma; VITAL Pakistan<br>Farooq, Fouzia; The George Washington University, Department of Global Health<br>Nisar, Muhammad; The Aga Khan University, Department of Pediatrics and Child Health<br>Jehan, Fyezah; The Aga Khan University, Department of Pediatrics and Child Health<br>Smith, Emily ; The George Washington University, Public Health<br>Hoodbhoy, Zahra; The Aga Khan University, Department of Pediatrics and Child Health
**Primary Subject Heading**:	Public health
Secondary Subject Heading:	Epidemiology, Paediatrics, Public health
Keywords:	Follow-Up Studies, Community child health < PAEDIATRICS, Primary Health Care, PUBLIC HEALTH, Risk Factors

SCHOLARONE™
Manuscripts

BMJ

I, the Submitting Author has the right to grant and does grant on behalf of all authors of the Work (as defined in the below author licence), an exclusive licence and/or a non-exclusive licence for contributions from authors who are: i) UK Crown employees; ii) where BMJ has agreed a CC-BY licence shall apply, and/or iii) in accordance with the terms applicable for US Federal Government officers or employees acting as part of their official duties; on a worldwide, perpetual, irrevocable, royalty-free basis to BMJ Publishing Group Ltd ("BMJ") its licensees and where the relevant Journal is co-owned by BMJ to the co-owners of the Journal, to publish the Work in this journal and any other BMJ products and to exploit all rights, as set out in our licence.

The Submitting Author accepts and understands that any supply made under these terms is made by BMJ to the Submitting Author unless you are acting as an employee on behalf of your employer or a postgraduate student of an affiliated institution which is paying any applicable article publishing charge ("APC") for Open Access articles. Where the Submitting Author wishes to make the Work available on an Open Access basis (and intends to pay the relevant APC), the terms of reuse of such Open Access shall be governed by a Creative Commons licence – details of these licences and which Creative Commons licence will apply to this Work are set out in our licence referred to above.

Other than as permitted in any relevant BMJ Author's Self Archiving Policies, I confirm this Work has not been accepted for publication elsewhere, is not being considered for publication elsewhere and does not duplicate material already published. I confirm all authors consent to publication of this Work and authorise the granting of this licence.

For peer review only

Title: Cohort profile: The Pregnancy Risk Infant Surveillance and Measurement Alliance (PRISMA) - Pakistan

Authors: Sabahat Naz[1], Ali Jaffar[1], Nida Yazdani[2], Muhammad Kashif[1], Zaid Hussain[1], Uzma Khan[2], Fouzia Farooq[3], Muhammad Imran Nisar[1], Fyezah Jehan[1], Emily R. Smith[3], Zahra Hoodbhoy[1]

[1]Department of Pediatrics and Child Health, The Aga Khan University, Stadium Road, Karachi, Pakistan 74800

[2]VITAL Pakistan Trust, Karachi, Pakistan

[3]Department of Global Health, Milken Institute School of Public Health, The George Washington University, Washington, D.C. 20052, USA

Corresponding author:

Zahra Hoodbhoy

Department of Pediatrics and Child Health

The Aga Khan University

Stadium Road, Karachi, Pakistan 74800

Tel No: 92-2134864126

Email: zahra.hoodbhoy@aku.edu

For peer review only

Abstract

Purpose:

Pakistan has disproportionately high maternal and neonatal morbidity and mortality. There is a lack of detailed, population-representative data to provide evidence for risk factors, morbidities, and mortality among pregnant women and their newborns. Pregnancy Risk, Infant Surveillance, and Measurement Alliance (PRISMA) is a multi-country open cohort that aims to collect high-dimensional, standardized data across five South Asian and African countries for estimating risk and developing innovative strategies to optimize pregnancy outcomes. This study presents the baseline maternal and neonatal characteristics of the Pakistan site.

Participants:

PRISMA Pakistan study is being conducted at two peri-urban field sites in Karachi, Pakistan. These sites have primary health care clinics where pregnant women and their newborns are followed during the antenatal, intrapartum, and postnatal periods up to one year after delivery. All encounters are captured electronically through a custom-built Android application. A total of 3,731 pregnant women with a mean age of 26.6 ± 5.8 years at the time of pregnancy with neonatal outcomes between January 2021 and August 2022 serve as a baseline for the PRISMA Pakistan study.

Findings to date:

In this cohort, live births accounted for most pregnancy outcomes (92%, n=3,478), followed by miscarriages/abortions (5.5%, n=205) and stillbirths (2.6%, n=98). Twenty-two percent of women (n=786) delivered at home. One out of every four neonates was low birth weight (< 2500 grams), and one out of every five was preterm (gestational age <37 weeks). The maternal mortality rate

For peer review only - http://bmjopen.bmj.com/site/about/guidelines.xhtml

was 172/100,000 pregnancies, the neonatal mortality rate was 52/1000 live births, and the stillbirth rate was 27/1000 births. The three most common causes of neonatal deaths obtained through verbal autopsy were perinatal asphyxia (39.6%), preterm births (19.8%), and infections (12.6%).

Future plans:

The PRISMA cohort will provide data-driven insights to prioritize and design interventions to improve maternal and neonatal outcomes in low-resource regions.

Trial registration number: NCT05904145

For peer review only

Strengths and limitations of this study

- This is a unique longitudinal cohort in Pakistan with the ability to leverage an existing demographic surveillance system to capture early pregnancies and follow these women postpartum with low attrition rates.
- As this cohort is situated in a peri-urban setting, it is representative of similar settings in low resource regions in South Asia.
- Although women are encouraged to deliver at the recommended referral facility for obtaining their information, nearly 36% delivered at other health facilities or their homes. In such cases, data on key labor and delivery outcomes is self-reported, thus limiting its interpretation.

For peer review only

INTRODUCTION:

Population-based longitudinal birth cohorts provide important data to study a life course approach to in-utero exposures and etiological factors that contribute to developing diseases early and later in life (1). The advantages of such cohort studies include standardized data collection, a systematic approach in following participants throughout the study, and the generalizability of findings to the larger population (1). In addition, it allows investigators to collect accurate information on the exposures, outcomes, potential confounders, and other important characteristics at the individual as well as population level (2). Early identification of these risk factors may provide an opportunity to design and implement population-based preventive strategies to optimize individual and population-level outcomes (3).

Establishing robust perinatal datasets in low-resource settings is essential for tracking local demographic shifts related to risk factors, morbidities and mortality outcomes among pregnant women and their newborns (4). This information can also aid in the development of stratified care pathways for efficient resource utilization thus improving outcomes (3). The Demographic Health Survey (DHS) and the Maternal Newborn Health Registry of the NICHD Global Network for Women's and Children's Health Research are examples that have generated a wealth of data over a decade in low and middle-income (LMICs) countries, including Pakistan (4, 5). However, these datasets are either cross-sectional (i.e., DHS), have limited timepoints during the pregnancy care continuum, and focus on maternal and neonatal mortality as primary outcomes with lack of detailed clinical and laboratory data on major morbidities in the mother and infant.

To address these gaps, the Pregnancy Risk Infant Surveillance and Measurement Alliance (PRISMA) Maternal and Newborn Health (MNH) study is being conducted across five sites in South Asia (India, Pakistan) and Sub-Saharan Africa (Zambia, Kenya, Ghana) to longitudinally assess pregnancy risk factors and their relationships with poor pregnancy outcomes, such as stillbirth, neonatal morbidity and mortality, and maternal mortality and severe maternal and infant morbidity. This multi-country, open cohort study aims to provide a harmonized dataset to quantify the burden of maternal, fetal, and neonatal risk factors and understand vulnerabilities, morbidity, and mortality.

The objective of this paper was to provide an overview of the baseline characteristics of women in the study catchment area for the Pakistan site before the initiation of the PRISMA study.

For peer review only

COHORT DESCRIPTION:

Study design and participants

In Pakistan, the PRISMA study will be conducted in two peri-urban community settings in Karachi, namely Rehri Goth and Ibrahim Hyderi; the areas comprise approximately 23,170 households and a population of 227,549 individuals (refer to Supplemental Figure 1).

The existing maternal and child health surveillance system at these sites will be leveraged for the early identification of pregnancies for the PRISMA cohort. The Department of Pediatrics and Child Health at the Aga Khan University has been conducting maternal and child surveillance at the peri-urban coastal field sites since 2010 (6). This system gathers data on vital events regarding married women of reproductive age and children under 5 years of age.

Pregnant women captured through pregnancy surveillance are encouraged to visit the primary health care (PHC) clinics located within the community for antenatal and postnatal care. Pregnant women aged 15 - 49 years with a confirmed intrauterine pregnancy of gestational age (GA) <20 weeks via standard ultrasound (GE Vivid IQ) are eligible to participate in the study. A total of 3,731 pregnant women with a neonatal outcome between January 2021 to August 2022 serve as a baseline for PRISMA Pakistan. The flow chart of this population is described in Figure 1.

Figure 1. Flow diagram for PRISMA enrollment status January 2021 to August 2022

Measures and procedure

At baseline, pregnant women visit the PHC located within the study catchment area for antenatal care services. This care is being offered by community midwives located at the designated centers. At the first antenatal care (ANC) visit, the pregnancy history of the women along with anthropometry, blood pressure and other vital information regarding the current pregnancy is obtained. The pregnant woman has her ultrasound to establish the GA at the first visit followed by laboratory investigations (including hemoglobin, hepatitis B and C screening). The subsequent ANC visit dates are given as per WHO recommendations for ANC visits (7). During delivery, women are provided transport to our partnering hospital to ensure skilled labor and delivery. In cases where women give birth at home or at another facility besides our partnering facility, community health workers (CHWs) visit the household to obtain information regarding labor and delivery, complications, if any, and perform newborn measurements (weight, length, and head circumference) within 72 hours of life. In case of a

For peer review only

maternal or infant mortality, a verbal autopsy is performed using the WHO 2016 verbal autopsy instrument (8) by trained research staff and coded by a physician to ascertain cause of death.

All visits are captured electronically through a custom-built Android application. This application uses an Open Smart Register Platform, an open-source mobile health platform designed to support the WHO SMART guidelines for data-driven decision-making in healthcare (9). Once entered into the application, data is extracted, transformed, and loaded into a PostgreSQL (10) relational database management system to allow for monitoring, reporting, and analytics. The application is user-specific and team-based (midwives, CHWs), with each team having a unique ID and password for data collection. The coordinators at the study sites perform period data checks and monitoring in order to ensure accurate data collection.

Patient and public involvement:

Written informed consent is obtained from all participants in the local language (Urdu). For illiterate participants, consent was obtained through a trusted witness who explained the consent form in the participant's language. A thumbprint or mark was taken from the participant, with both the participant and the witness signing the form to validate the process. The cohort participants are not involved in the design or recruitment of the study. However, the community mobilization teams of PRISMA Pakistan have been actively engaged with community stakeholders and pregnant women to inform on the study and benefits of early initiation of antenatal care.

Ethical Approval:

The PRISMA MNH Pakistan baseline study received ethical approval in Pakistan (001-VPT-IRB-20).

FINDINGS TO DATE:

As of August 2022, 3,731 women with pregnancy outcomes serve as a baseline for the PRISMA Pakistan cohort (Table 2). The sociodemographic and clinical characteristics of women included, as well as those excluded from the study, are described in Table 1.

For peer review only

Table 1. Sociodemographic and clinical characteristics of women included and excluded in the baseline cohort of PRISMA Pakistan

Characteristics	Women included in the analysis (n = 3,731)<br><br>Mean ± SD, Median (range) or n (%)	Women who did not receive ANC services or did not consent (n= 5,714)<br><br>Mean ± SD, Median (range) or n (%)
Age at marriage (years)	20.1 ± 3.2	28.2 ± 6.1
**Maternal education**		
Literate	1,198 (33.1)	1,934 (38.4)
**Women's employment status**		
Employed	1,129 (31.2)	-
**Husband's employment status**		
Employed	3,582 (98.9)	-
**Wealth Index (quintiles)**		-
Q1 (least wealthy)	791 (21.8)	
Q2	659 (18.2)	
Q3	736 (20.3)	
Q4	813 (22.4)	
Q5 (wealthiest)	624 (17.2)	
Reported Smoking	95 (2.6)	-
Current use of smokeless tobacco	818 (21.9)	-
Total number of pregnancies	3 (1 - 16)	3 (1 – 20)
Primigravida	836 (22.4)	826 (16.3)
Multigravida (2 – 5)	2,232 (59.8)	2,576 (51)
Grand Multigravida (>5)	663 (17.8)	1,654 (32.7)
No. of previous livebirths	2 (1 - 14)	3 (1 – 15)
No. of previous stillbirths	1 (1 - 5)	1 (1 – 9)
No. of previous miscarriages	1 (1 - 10)	1 (1 – 9)
No. of previous induced abortions	1 (1 - 3)	1 (1 – 5)

For peer review only

Women enrolled in this cohort have a mean age of 26.6 ± 5.8 years at the time of pregnancy. The average number of antenatal visits were 5.4 ± 2.8, with a mean GA of 17.2 ± 8.1 weeks at the first visit. Using the cutoff of mid-upper arm circumference (MUAC) of <23 cm, 27% (n=1,006) of pregnant were malnourished. Approximately two-thirds of women

For peer review only - http://bmjopen.bmj.com/site/about/guidelines.xhtml

(64%, n=2570) had either mild, moderate, or severe anemia. Detailed pregnancy characteristics of these women are described in Table 2.

Table 2. Pregnancy characteristics of enrolled participants

Characteristics	Total Population (n = 3,731) Mean ± SD; n (%)
Age at the time of pregnancy (years)	26.6 ± 5.8
Number of ANC visits	5.4 ± 2.8
GA at first ANC visit (weeks)	17.2 ± 8.1
MUAC* (cm)	25.8 ± 4.1
**Nutritional status based on MUAC (cm)**	
Normal (≥23)	2,724 (73.0)
Malnourished (<23)	1,006 (27.0)
BMI (kg/m^2)	23.4 ± 5.2
**Nutritional status based on BMI (kg/m^2)**	
Underweight (<18.5)	564 (15.1)
Normal weight (18.5 – 22.9)	1,454 (39.0)
Overweight (23.0 – <26.9)	891 (23.9)
Obese (≥27.0)	821 (22.0)
Hemoglobin levels (g/dL)	10.3 ± 1.4
**Anemia status (hemoglobin level)**	
Normal (≥11.0 g/dL)	1,161 (35.7)
Mild Anemia (10.0 - 10.9 g/dL)	940 (28.9)
Moderate Anemia (7.0 - 9.9 g/dL)	1,077 (33.1)
Severe Anemia (<7.0 g/dL)	71 (2.2)
Chronic Hypertension	124 (3.3)
Diabetes Mellitus	23 (0.6)
Gestational Hypertension	48 (1.3)
Hepatitis B	33 (1.2)
Hepatitis C	52 (1.9)

BMI, Body mass index; GA, Gestational age; MUAC, Mid-upper arm circumference

For peer review only

Majority of the pregnancy outcomes in this cohort were live births (92%, n=3,478) followed by miscarriages/abortions (5.5%, n=205) and stillbirths (2.6%, n=98). Twenty-two percent (n=786) of these women delivered at home. Overall, 20.1% (n=696) infants were preterm, and 24.1% (n=611) were low birthweight (Table 3).

Table 3. Delivery characteristics and neonatal outcomes in the study population

Characteristics	Total Population (n = 3,576) n (%)
**Number of fetuses**	
Single	3,618 (98.7)
Twins	48 (1.3)
**Birth outcomes**	
Livebirths	3,478 (92.0)
Stillbirths	98 (2.6)
Miscarriages	180 (4.8)
Abortions	25 (0.7)
**Gender**	
Male	1,836 (51.3)
Female	1,738 (48.6)
**Place of delivery**	
Partner referral facility	2,288 (64.0)
Other facilities	502 (14.0)
Home births	786 (22.0)
**Mode of delivery**	
Vaginal	2,620 (73.3)
Spontaneous	2617 (99.9)
Assisted	3 (0.1)
Cesarean	956 (26.7)
Elective	530 (55.7)
Emergent	422 (44.3)
**Gestational age at delivery (in weeks)**	
Extremely preterm (<28)	8 (0.2)
Very preterm (28 - <32)	44 (1.3)
Moderate-to-late preterm (32 - < 37)	644 (18.6)
Term (≥37)	2,761 (79.9)
**Birth weight categories (in kg)**	
Extremely low birth weight (<1)	2 (0.1)
Very low birth weight (1 – < 1.5)	31 (1.2)
Low birth weight (1.5 - <2.5)	578 (22.8)
Normal birth weight (≥2.5)	1,930 (75.9)

For peer review only

For peer review only - http://bmjopen.bmj.com/site/about/guidelines.xhtml

There were 98 stillbirths among this cohort, with 75% intrapartum stillbirths (n=74) and 25% antepartum stillbirths (n=24). Nearly three-quarters of neonatal deaths (76.4%, n=139) occurred during the first week of life. The three main causes of death during the neonatal period were perinatal asphyxia (39.6%, n=72), preterm birth (19.8%, n=36), and infections (12.6%, n=23).

Maternal and neonatal mortality rates are described in Table 4. In this cohort, the maternal mortality rate is 172 per 100,000 live births, which is lower than the national maternal mortality (186 per 100,000 live births) (11). Rate of stillbirths in this cohort is 27 per 1000 births, which is also lower than the national rate of 44 per 1,000 live births (12). On the other hand, the neonatal mortality rates in the PRISMA Pakistan cohort (52 per 1,000 live births) are comparable with the national neonatal mortality rates (51 per 1,000 livebirths) (12). The lower rates of maternal mortality and stillbirth observed in this cohort may be attributed to the provisions of adequate antenatal care as well as timely referrals when needed. However, postnatal care primarily takes place at home, and the lack of follow-up could potentially explain the higher neonatal mortality.

Table 4. Rates of maternal and neonatal mortality and stillbirth in PRISMA Pakistan

Indicators	Mortality rates with (95% CI) in the study cohort	Mortality rates in Pakistan (2018-19) (11, 12)
**Maternal mortality rate**	172 (63, 375)/100,000 livebirths	186 /100,000 livebirths
**Stillbirth rate**	27 (22, 33)/1,000 births	44/1,000 births
**Neonatal mortality rate**	52 (45, 60)/1,000 livebirths	51/1,000 livebirths

STRENGTHS AND LIMITATIONS OF THIS STUDY

Since this cohort leverages the community-based setting, the population included in this study is a good representation of those residing in similar low-resource settings. The existing demographic surveillance system is leveraged to capture pregnancies and follow pregnant women and infants for up to one year after delivery. However, there are certain limitations with this work. Currently, women seek antenatal care in the second trimester. Our estimates regarding early-pregnancy outcomes, such as miscarriage/abortion, are underestimated given the mean gestational age at enrollment of 17 weeks. For the next phase of the PRISMA study, community mobilization efforts will be put in place so that majority of the women

For peer review only - http://bmjopen.bmj.com/site/about/guidelines.xhtml

enroll in the first trimester of pregnancy. Information on some key variables, such as pregnancy history as well as labor and delivery outcomes for those women who delivered at home and outside the recommended referral facility is self-reported, thus limiting its interpretation. Another limitation is that not all women from the community are seeking care during pregnancy, and hence may be different from those enrolled in PRISMA Pakistan, thus limiting the generalizability. This phenomenon has also been reported in other studies where women who do not seek ANC services or seek services later in their pregnancy maybe more disadvantaged in terms of their socio-demographic characteristics (13, 14), thus predisposing them to poorer outcomes (15). However, with the existing baseline data acquired through the demographic surveillance system, the study team will be able to compare any differences in health-seeking behaviors in order to make the study findings more generalizable.

COLLABORATION

The study protocol for the next phase of this study, a harmonized multi-site PRISMA open cohort is registered with https://clinicaltrials.gov (number: NCT05904145) or by contacting the corresponding author of this manuscript.

Acknowledgments: The authors acknowledge the contributions of all women who participated in the study.

Contributors:

ZH, FJ, MIN, FF, and ES conceptualized the study and secure funding. ZH, NY, and UK supervised the study at field sites. ZH, SN, AJ, NY, MK, ZHU, and UK were involved in the analysis, interpretation, and writing manuscript. ZH, FJ, MIN, FF, and ES reviewed and provided scientific revision to the manuscript. All authors agreed prior to submission to take responsibility and be accountable for the contents of the manuscript. All authors read and approved the final manuscript.

Funding Statement: This study is funded by the Bill & Melinda Gates Foundation (INV-005776**).**

Competing Interests: None declared

For peer review only

References:

1. Manolio TA, Bailey-Wilson JE, Collins FS. Genes, environment and the value of prospective cohort studies. Nat Rev Genet. 2006;7(10):812-20.
2. Lawlor DA, Andersen AM, Batty GD. Birth cohort studies: past, present and future. Int J Epidemiol. 2009;38(4):897-902.
3. Baqui A, Ahmed P, Dasgupta SK, Begum N, Rahman M, Islam N, et al. Development and validation of a simplified algorithm for neonatal gestational age assessment - protocol for the Alliance for Maternal Newborn Health Improvement (AMANHI) prospective cohort study. J Glob Health. 2017;7(2):021201.
4. Patel KK, Rai R, Rai AK. Determinants of infant mortality in Pakistan: evidence from Pakistan Demographic and Health Survey 2017–18. Journal of Public Health. 2021;29:693-701.
5. McClure EM, Garces AL, Hibberd PL, Moore JL, Goudar SS, Saleem S, et al. The Global Network Maternal Newborn Health Registry: a multi-country, community-based registry of pregnancy outcomes. Reprod Health. 2020;17(Suppl 2):184.
6. Ilyas M, Naeem K, Fatima U, Nisar MI, Kazi AM, Jehan F, et al. Profile: health and demographic surveillance system in peri-urban areas of Karachi, Pakistan. Gates Open Research. 2018.
7. Organization WH. WHO recommendations on antenatal care for a positive pregnancy experience: World Health Organization; 2016.
8. Nichols EK, Byass P, Chandramohan D, Clark SJ, Flaxman AD, Jakob R, et al. The WHO 2016 verbal autopsy instrument: An international standard suitable for automated analysis by InterVA, InSilicoVA, and Tariff 2.0. PLoS medicine. 2018;15(1):e1002486.
9. A world free from hunger and all forms of malnutrition is attainable in this generation: United Nations System Standing Committee on Nutrition [Available from: https://www.unscn.org/en/topics/nutrition_and_crises.
10. Momjian B. PostgreSQL Introduction and Concepts Bruce Momjian ADDISON. 2001.
11. Azam Basti is part of Jamshed Town: Government of Karachi; [updated 27 May 2022. Available from: https://en.wikipedia.org/wiki/Azam_Basti.
12. Aziz A, Saleem S, Nolen TL, Pradhan NA, McClure EM, Jessani S, et al. Why are the Pakistani maternal, fetal and newborn outcomes so poor compared to other low and middle-income countries? Reprod Health. 2020;17(Suppl 3):190.
13. Nisar YB, Aurangzeb B, Dibley MJ, Alam A. Qualitative exploration of facilitating factors and barriers to use of antenatal care services by pregnant women in urban and rural settings in Pakistan. BMC pregnancy and childbirth. 2016;16:1-9.
14. Agha S, Tappis H. The timing of antenatal care initiation and the content of care in Sindh, Pakistan. BMC pregnancy and childbirth. 2016;16:1-9.
15. Alwan NA, Roderick PJ, Macklon NS. Is timing of the first antenatal visit associated with adverse birth outcomes? Analysis from a population-based birth cohort. The Lancet. 2016;388:S18.

For peer review only

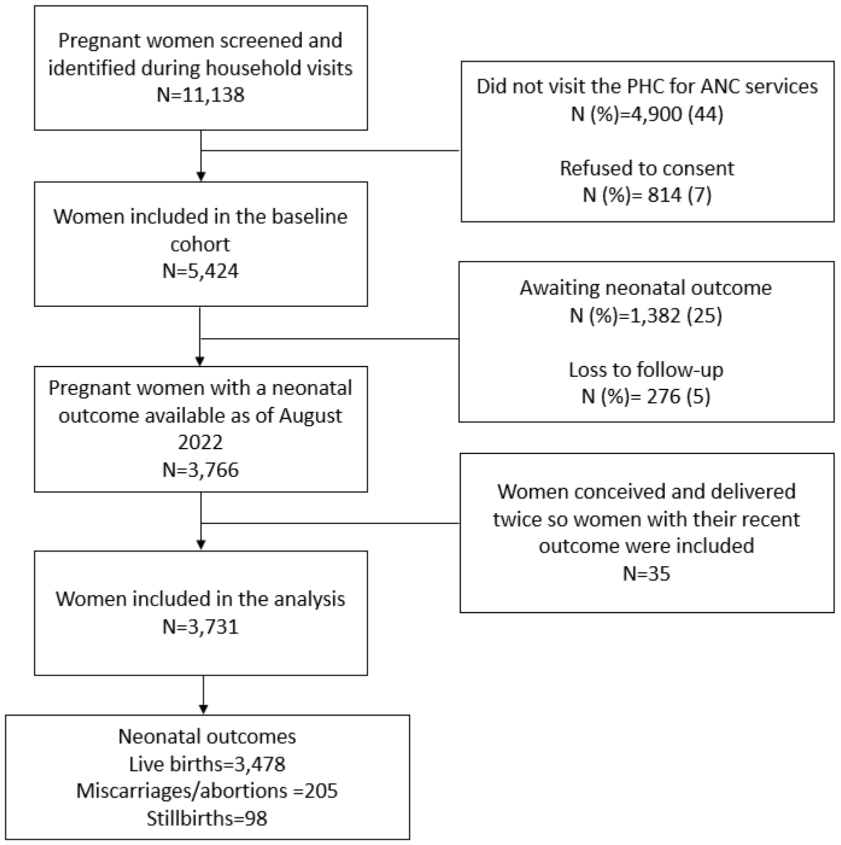

PHC= Primary healthcare, ANC= Antenatal care

Supplemental Figure 1. PRISMA study sites in Karachi, Pakistan

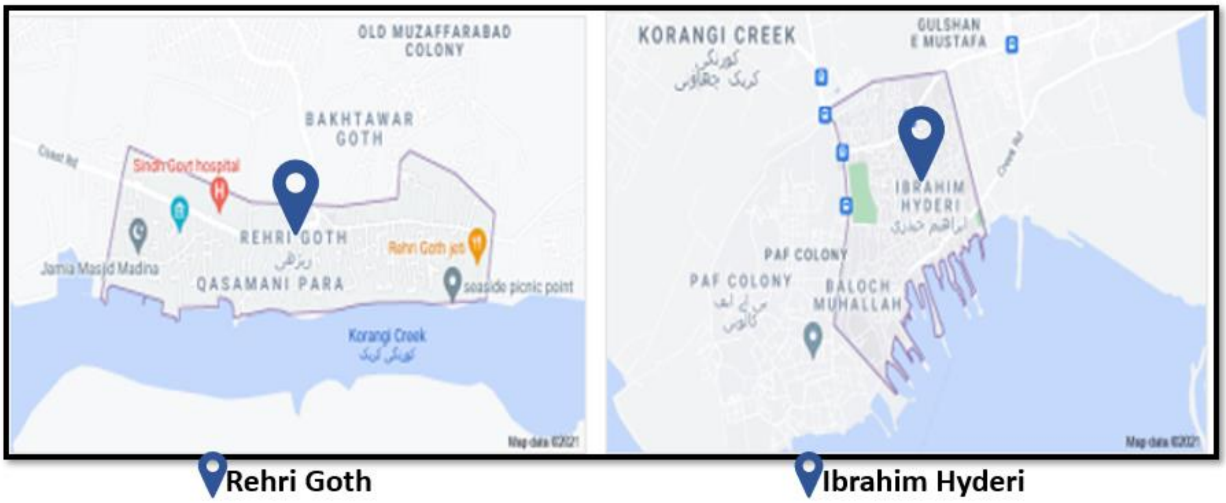

For peer review only

STROBE Statement—Checklist of items that should be included in reports of ***cohort studies***

	Item No	Recommendation	Page No
**Title and abstract**	1	(*a*) Indicate the study's design with a commonly used term in the title or the abstract	1
		(*b*) Provide in the abstract an informative and balanced summary of what was done and what was found	2-3
**Introduction**			
Background/rationale	2	Explain the scientific background and rationale for the investigation being reported	5
Objectives	3	State specific objectives, including any prespecified hypotheses	5
**Methods**			
Study design	4	Present key elements of study design early in the paper	6
Setting	5	Describe the setting, locations, and relevant dates, including periods of recruitment, exposure, follow-up, and data collection	6
Participants	6	(*a*) Give the eligibility criteria, and the sources and methods of selection of participants. Describe methods of follow-up	6
		(*b*) For matched studies, give matching criteria and number of exposed and unexposed	NA
Variables	7	Clearly define all outcomes, exposures, predictors, potential confounders, and effect modifiers. Give diagnostic criteria, if applicable	NA
Data sources/ measurement	8*	For each variable of interest, give sources of data and details of methods of assessment (measurement). Describe comparability of assessment methods if there is more than one group	7-9
Bias	9	Describe any efforts to address potential sources of bias	NA
Study size	10	Explain how the study size was arrived at	NA
Quantitative variables	11	Explain how quantitative variables were handled in the analyses. If applicable, describe which groupings were chosen and why	NA
Statistical methods	12	(*a*) Describe all statistical methods, including those used to control for confounding	NA
		(*b*) Describe any methods used to examine subgroups and interactions	NA
		(*c*) Explain how missing data were addressed	NA
		(*d*) If applicable, explain how loss to follow-up was addressed	NA
		(*e*) Describe any sensitivity analyses	NA
**Results**			
Participants	13*	(a) Report numbers of individuals at each stage of study—eg numbers potentially eligible, examined for eligibility, confirmed eligible, included in the study, completing follow-up, and analysed	6
		(b) Give reasons for non-participation at each stage	NA
		(c) Consider use of a flow diagram	6
Descriptive data	14*	(a) Give characteristics of study participants (eg demographic, clinical, social) and information on exposures and potential confounders	7
		(b) Indicate number of participants with missing data for each variable of interest	NA
		(c) Summarise follow-up time (eg, average and total amount)	8-9
Outcome data	15*	Report numbers of outcome events or summary measures over time	NA

For peer review only

For peer review only - http://bmjopen.bmj.com/site/about/guidelines.xhtml

Main results	16	(*a*) Give unadjusted estimates and, if applicable, confounder-adjusted estimates and their precision (eg, 95% confidence interval). Make clear which confounders were adjusted for and why they were included	NA
		(*b*) Report category boundaries when continuous variables were categorized	NA
		(*c*) If relevant, consider translating estimates of relative risk into absolute risk for a meaningful time period	NA
Other analyses	17	Report other analyses done—eg analyses of subgroups and interactions, and sensitivity analyses	NA
**Discussion**			
Key results	18	Summarise key results with reference to study objectives	9-12
Limitations	19	Discuss limitations of the study, taking into account sources of potential bias or imprecision. Discuss both direction and magnitude of any potential bias	12-13
Interpretation	20	Give a cautious overall interpretation of results considering objectives, limitations, multiplicity of analyses, results from similar studies, and other relevant evidence	9-12
Generalisability	21	Discuss the generalisability (external validity) of the study results	12
**Other information**			
Funding	22	Give the source of funding and the role of the funders for the present study and, if applicable, for the original study on which the present article is based	13

*Give information separately for exposed and unexposed groups.

Note: An Explanation and Elaboration article discusses each checklist item and gives methodological background and published examples of transparent reporting. The STROBE checklist is best used in conjunction with this article (freely available on the Web sites of PLoS Medicine at http://www.plosmedicine.org/, Annals of Internal Medicine at http://www.annals.org/, and Epidemiology at http://www.epidem.com/). Information on the STROBE Initiative is available at http://www.strobe-statement.org.

For peer review only

For peer review only - http://bmjopen.bmj.com/site/about/guidelines.xhtml

BMJ Open

Cohort profile: The Pregnancy Risk Infant Surveillance and Measurement Alliance (PRISMA) - Pakistan

Journal:	*BMJ Open*
Manuscript ID	bmjopen-2023-078222.R2
Article Type:	Cohort profile
Date Submitted by the Author:	17-Nov-2023
Complete List of Authors:	Naz, Sabahat; The Aga Khan University, Department of Pediatrics and Child Health<br>Jaffar, Ali; The Aga Khan University, Department of Pediatrics and Child Health<br>Yazdani, Nida; VITAL Pakistan<br>Kashif, Muhammad ; The Aga Khan University, Department of Pediatrics and Child Health<br>Hussain, Zaid; The Aga Khan University, Department of Pediatrics and Child Health<br>Khan, Uzma; VITAL Pakistan<br>Farooq, Fouzia; The George Washington University, Department of Global Health<br>Nisar, Muhammad; The Aga Khan University, Department of Pediatrics and Child Health<br>Jehan, Fyezah; The Aga Khan University, Department of Pediatrics and Child Health<br>Smith, Emily ; The George Washington University, Public Health<br>Hoodbhoy, Zahra; The Aga Khan University, Department of Pediatrics and Child Health
**Primary Subject Heading**:	Public health
Secondary Subject Heading:	Epidemiology, Paediatrics, Public health
Keywords:	Follow-Up Studies, Community child health < PAEDIATRICS, Primary Health Care, PUBLIC HEALTH, Risk Factors

SCHOLARONE™
Manuscripts

BMJ

I, the Submitting Author has the right to grant and does grant on behalf of all authors of the Work (as defined in the below author licence), an exclusive licence and/or a non-exclusive licence for contributions from authors who are: i) UK Crown employees; ii) where BMJ has agreed a CC-BY licence shall apply, and/or iii) in accordance with the terms applicable for US Federal Government officers or employees acting as part of their official duties; on a worldwide, perpetual, irrevocable, royalty-free basis to BMJ Publishing Group Ltd ("BMJ") its licensees and where the relevant Journal is co-owned by BMJ to the co-owners of the Journal, to publish the Work in this journal and any other BMJ products and to exploit all rights, as set out in our licence.

The Submitting Author accepts and understands that any supply made under these terms is made by BMJ to the Submitting Author unless you are acting as an employee on behalf of your employer or a postgraduate student of an affiliated institution which is paying any applicable article publishing charge ("APC") for Open Access articles. Where the Submitting Author wishes to make the Work available on an Open Access basis (and intends to pay the relevant APC), the terms of reuse of such Open Access shall be governed by a Creative Commons licence – details of these licences and which Creative Commons licence will apply to this Work are set out in our licence referred to above.

Other than as permitted in any relevant BMJ Author's Self Archiving Policies, I confirm this Work has not been accepted for publication elsewhere, is not being considered for publication elsewhere and does not duplicate material already published. I confirm all authors consent to publication of this Work and authorise the granting of this licence.

For peer review only

Title: Cohort profile: The Pregnancy Risk Infant Surveillance and Measurement Alliance (PRISMA) - Pakistan

Authors: Sabahat Naz[1], Ali Jaffar[1], Nida Yazdani[2], Muhammad Kashif[1], Zaid Hussain[1], Uzma Khan[2], Fouzia Farooq[3], Muhammad Imran Nisar[1], Fyezah Jehan[1], Emily R. Smith[3], Zahra Hoodbhoy[1]

[1]Department of Pediatrics and Child Health, The Aga Khan University, Stadium Road, Karachi, Pakistan 74800

[2]VITAL Pakistan Trust, Karachi, Pakistan

[3]Department of Global Health, Milken Institute School of Public Health, The George Washington University, Washington, D.C. 20052, USA

Corresponding author:

Zahra Hoodbhoy

Department of Pediatrics and Child Health

The Aga Khan University

Stadium Road, Karachi, Pakistan 74800

Tel No: 92-2134864126

Email: zahra.hoodbhoy@aku.edu

For peer review only

Abstract

Purpose:

Pakistan has disproportionately high maternal and neonatal morbidity and mortality. There is a lack of detailed, population-representative data to provide evidence for risk factors, morbidities, and mortality among pregnant women and their newborns. The Pregnancy Risk, Infant Surveillance, and Measurement Alliance (PRISMA) is a multi-country open cohort that aims to collect high-dimensional, standardized data across five South Asian and African countries for estimating risk and developing innovative strategies to optimize pregnancy outcomes for mothers and their newborns. This study presents the baseline maternal and neonatal characteristics of the Pakistan site occurring prior to the launch of a multi-site, harmonized protocol.

Participants:

PRISMA Pakistan study is being conducted at two peri-urban field sites in Karachi, Pakistan. These sites have primary health care clinics where pregnant women and their newborns are followed during the antenatal, intrapartum, and postnatal periods up to one year after delivery. All encounters are captured electronically through a custom-built Android application. A total of 3,731 pregnant women with a mean age of 26.6 ± 5.8 years at the time of pregnancy with neonatal outcomes between January 2021 and August 2022 serve as a baseline for the PRISMA Pakistan study.

Findings to date:

In this cohort, live births accounted for the majority of pregnancy outcomes (92%, n=3,478), followed by miscarriages/abortions (5.5%, n=205) and stillbirths (2.6%, n=98). Twenty-two percent of women (n=786) delivered at home. One out of every four neonates was low birth weight

For peer review only

(< 2500 grams), and one out of every five was preterm (gestational age <37 weeks). The maternal mortality rate was 172/100,000 pregnancies, the neonatal mortality rate was 52/1000 live births, and the stillbirth rate was 27/1000 births. The three most common causes of neonatal deaths obtained through verbal autopsy were perinatal asphyxia (39.6%), preterm births (19.8%), and infections (12.6%).

Future plans:

The PRISMA cohort will provide data-driven insights to prioritize and design interventions to improve maternal and neonatal outcomes in low-resource regions.

Trial registration number: NCT05904145

For peer review only

For peer review only - http://bmjopen.bmj.com/site/about/guidelines.xhtml

Strengths and limitations of this study

- The prospective nature of the PRISMA Pakistan cohort enables the collection of a population-based, high-quality, clinical cohort of pregnancies within the defined geographical area.
- This is a unique population-based, longitudinal cohort in Pakistan with the ability to leverage an existing demographic surveillance system to capture early pregnancies and follow these women postpartum with low attrition rates.
- Although women are encouraged to deliver at the recommended referral facility for obtaining their information, nearly 36% delivered at other health facilities or their homes. In such cases, data on key labor and delivery outcomes is self-reported, thus limiting its interpretation.

For peer review only

For peer review only - http://bmjopen.bmj.com/site/about/guidelines.xhtml

INTRODUCTION:

Population-based longitudinal birth cohorts provide important data to study a life course approach to in-utero exposures and etiological factors that contribute to developing diseases early and later in life (1). The advantages of such cohort studies include standardized data collection, a systematic approach in following participants throughout the study, and the generalizability of findings to the larger population (1). In addition, it allows investigators to collect accurate information on the exposures, outcomes, potential confounders, and other important characteristics at the individual as well as population level (2). Early identification of these risk factors may provide an opportunity to design and implement population-based preventive strategies to optimize individual and population-level outcomes (3).

Establishing robust perinatal datasets in low-resource settings is essential for tracking local demographic shifts related to risk factors, morbidities and mortality outcomes among pregnant women and their newborns (4). This information can also aid in the development of stratified care pathways for efficient resource utilization, thus improving outcomes (3). The Demographic Health Survey (DHS) and the Maternal Newborn Health Registry of the NICHD Global Network for Women's and Children's Health Research are examples that have generated a wealth of data over a decade in low and middle-income (LMICs) countries, including Pakistan (4, 5). However, these datasets are either cross-sectional (i.e., DHS) or have limited timepoints during the pregnancy care continuum. Furthermore, these datasets focus on maternal and neonatal mortality as primary outcomes and lack detailed clinical and laboratory data on major morbidities in the mother and infant.

To address these gaps, the Pregnancy Risk Infant Surveillance and Measurement Alliance (PRISMA) Maternal and Newborn Health (MNH) study is being conducted across five sites in South Asia (India, Pakistan) and Sub-Saharan Africa (Zambia, Kenya, Ghana) to longitudinally assess pregnancy risk factors and their relationships with poor pregnancy outcomes, such as stillbirth, neonatal morbidity and mortality, and maternal mortality and severe maternal and infant morbidity. This multi-country, open cohort study aims to provide a harmonized dataset to quantify the burden of maternal, fetal, and neonatal risk factors and understand vulnerabilities, morbidity, and mortality.

The objective of this paper was to provide an overview of the baseline characteristics of women in the study catchment area for the Pakistan site before the initiation of the PRISMA study.

For peer review only - http://bmjopen.bmj.com/site/about/guidelines.xhtml

COHORT DESCRIPTION:

Study design and participants

In Pakistan, the PRISMA study is being conducted in two peri-urban community settings in Karachi, namely Rehri Goth and Ibrahim Hyderi; the areas comprise approximately 23,170 households and a population of 227,549 individuals (refer to Supplemental Figure 1).

The existing maternal and child health surveillance system at these sites will be leveraged for the early identification of pregnancies for the PRISMA cohort. The Department of Pediatrics and Child Health at the Aga Khan University (AKU) has been conducting maternal and child surveillance at the peri-urban coastal field sites since 2003 (6). This system gathers data on vital events regarding married women of reproductive age and children under five years of age. Records of births, deaths, pregnancies, and migration events are documented through quarterly household visits by Community Health Workers (CHWs) using the existing household rosters of these sites.

Pregnant women captured through pregnancy surveillance are encouraged to visit the primary health care (PHC) clinics located within the community for antenatal care. These PHCs are run by AKU and are not part of the public health system. Antenatal care, along with laboratory and ultrasound procedures, is offered free of cost for all pregnant women from the catchment area by community midwives located at these centers. Pregnant women aged 15 - 49 years with a confirmed intrauterine pregnancy of gestational age (GA) <20 weeks via standard ultrasound (GE Vivid IQ) (7) are eligible to participate in the study. A total of 3,731 pregnant women with a neonatal outcome of 3781 between January 2021 to August 2022 serve as a baseline for PRISMA Pakistan. The flow chart of this population is described in Figure 1.

Figure 1. Flow diagram for PRISMA enrollment status January 2021 to August 2022

Measures and procedure

At baseline, pregnant women visit the PHC located within the study catchment area for antenatal care services. At the first antenatal care (ANC) visit, the pregnancy history of the women along with anthropometry, blood pressure and other vital information regarding the current pregnancy is obtained. The pregnant woman has her ultrasound to establish the GA at the first visit followed by laboratory investigations (including hemoglobin, hepatitis B and C screening). The subsequent ANC visit dates are given as per WHO recommendations for ANC visits (8). During delivery, women are provided transport to our partnering hospital to ensure skilled labor and delivery. In cases where women give birth at home or at another facility

For peer review only

besides our partnering facility, community health workers (CHWs) visit the household to obtain information regarding labor and delivery, complications, if any, and perform newborn measurements (weight, length, and head circumference) within 72 hours of life. In case of a maternal or neonatal mortality, a verbal autopsy is performed using the WHO 2016 verbal autopsy instrument (9) by trained research staff and coded by a physician to ascertain cause of death.

All visits are captured electronically through a custom-built Android application. This application uses an Open Smart Register Platform, an open-source mobile health platform designed to support the WHO SMART guidelines for data-driven decision-making in healthcare (10). Once entered into the application, data is extracted, transformed, and loaded into a PostgreSQL (11) relational database management system to allow for monitoring, reporting, and analytics. The application is user-specific and team-based (midwives, CHWs), with each team having a unique ID and password for data collection. The coordinators at the study sites perform period data checks and monitoring in order to ensure accurate data collection.

Patient and public involvement:

Written informed consent is obtained from all participants in the local language (Urdu). For illiterate participants, consent was obtained through a trusted witness who explained the consent form in the participant's language. A thumbprint or mark was taken from the participant, with both the participant and the witness signing the form to validate the process. The cohort participants are not involved in the design or recruitment of the study. However, the community mobilization teams of PRISMA Pakistan have been actively engaged with community stakeholders and pregnant women to inform on the study and benefits of early initiation of antenatal care.

Ethical Approval:

The PRISMA MNH Pakistan baseline study received ethical approval in Pakistan (001-VPT-IRB-20).

FINDINGS TO DATE:

As of August 2022, 3,731 women with pregnancy outcomes serve as a baseline for the PRISMA Pakistan cohort (Table 2). The sociodemographic and clinical characteristics of women included, as well as those excluded from the study, are described in Table 1.

For peer review only - http://bmjopen.bmj.com/site/about/guidelines.xhtml

Table 1. Sociodemographic and clinical characteristics of women included and excluded in the baseline cohort of PRISMA Pakistan

Characteristics	Women included in the analysis (n = 3,731) Mean ± SD, Median (range) or n (%)	Women who did not receive ANC services or did not consent (n= 5,714) Mean ± SD, Median (range) or n (%)
Age at marriage (years)	20.1 ± 3.2	28.2 ± 6.1
**Maternal education**		
Literate	1,198 (33.1)	1,934 (38.4)
**Women's employment status**		
Employed	1,129 (31.2)	-
**Husband's employment status**		
Employed	3,582 (98.9)	-
**Wealth Index (quintiles)**		-
Q1 (least wealthy)	791 (21.8)	
Q2	659 (18.2)	
Q3	736 (20.3)	
Q4	813 (22.4)	
Q5 (wealthiest)	624 (17.2)	
Reported Smoking	95 (2.6)	-
Current use of smokeless tobacco	818 (21.9)	-
Total number of pregnancies	3 (1 - 16)	3 (1 – 20)
Primigravida	836 (22.4)	826 (16.3)
Multigravida (2 – 5)	2,232 (59.8)	2,576 (51)
Grand Multigravida (>5)	663 (17.8)	1,654 (32.7)
No. of previous livebirths	2 (1 - 14)	3 (1 – 15)
No. of previous stillbirths	1 (1 - 5)	1 (1 – 9)
No. of previous miscarriages	1 (1 - 10)	1 (1 – 9)
No. of previous induced abortions	1 (1 - 3)	1 (1 – 5)

For peer review only

Women enrolled in this cohort have a mean age of 26.6 ± 5.8 years at the time of pregnancy. The average number of antenatal visits were 5.4 ± 2.8, with a mean GA of 17.2 ± 8.1 weeks at the first visit. Using the cutoff of mid-upper arm circumference (MUAC) of <23 cm, 27% (n=1,006) of pregnant were malnourished. Approximately two-thirds of women (64%, n=2570) had either mild, moderate, or severe anemia. Detailed pregnancy characteristics of these women are described in Table 2.

Table 2. Pregnancy characteristics of enrolled participants

Characteristics	Total Population (n = 3,731)<br>Mean ± SD; n (%)
Age at the time of pregnancy (years)	26.6 ± 5.8
Number of ANC visits	5.4 ± 2.8
GA at first ANC visit (weeks)	17.2 ± 8.1
MUAC* (cm)	25.8 ± 4.1
**Nutritional status based on MUAC (cm)**	
Normal (≥23)	2,724 (73.0)
Malnourished (<23)	1,006 (27.0)
BMI (kg/m^2)	23.4 ± 5.2
**Nutritional status based on BMI (kg/m^2)**	
Underweight (<18.5)	564 (15.1)
Normal weight (18.5 – 22.9)	1,454 (39.0)
Overweight (23.0 – <26.9)	891 (23.9)
Obese (≥27.0)	821 (22.0)
Hemoglobin levels (g/dL)	10.3 ± 1.4
**Anemia status (hemoglobin level)**	
Normal (≥11.0 g/dL)	1,161 (35.7)
Mild Anemia (10.0 - 10.9 g/dL)	940 (28.9)
Moderate Anemia (7.0 - 9.9 g/dL)	1,077 (33.1)
Severe Anemia (<7.0 g/dL)	71 (2.2)
Chronic Hypertension	124 (3.3)
Diabetes Mellitus	23 (0.6)
Gestational Hypertension	48 (1.3)
Hepatitis B	33 (1.2)
Hepatitis C	52 (1.9)

BMI, Body mass index; GA, Gestational age; MUAC, Mid-upper arm circumference

For peer review only - http://bmjopen.bmj.com/site/about/guidelines.xhtml

Majority of the pregnancy outcomes in this cohort were live births (92%, n=3,478) followed by miscarriages/abortions (5.5%, n=205) and stillbirths (2.6%, n=98). Twenty-two percent (n=786) of these women delivered at home. Overall, 20.1% (n=696) infants were preterm, and 24.1% (n=611) were low birthweight (Table 3).

Table 3. Delivery characteristics and neonatal outcomes in the study population

Characteristics	n (%)
**Number of Pregnant Women**	**3,731**
**Number of fetuses**	
Single	3,681 (98.7)
Multiple gestation	50 (1.3)
**Place of delivery**[a]	
Partner referral facility	2,288 (64.0)
Other facilities	502 (14.0)
Home births	786 (22.0)
**Mode of delivery**[a]	
Vaginal	2,620 (73.3)
Spontaneous	2617 (99.9)
Assisted	3 (0.1)
Cesarean	956 (26.7)
Elective	530 (55.7)
Emergent	422 (44.3)
**Number of Birth Outcomes**	**3781**
**Birth outcomes**[b]	
Livebirths	3,478 (92.0)
Stillbirths	98 (2.6)
Miscarriages	180 (4.8)
Abortions	25 (0.7)
**Gender**[a]	
Male	1,836 (51.3)
Female	1,738 (48.6)
Ambiguous	2 (0.0)
**Gestational age at delivery (weeks)**[c]	
Extremely preterm (<28)	8 (0.2)
Very preterm (28 - <32)	44 (1.3)
Moderate-to-late preterm (32 - < 37)	644 (18.6)
Term (≥37)	2,761 (79.9)
**Birth weight categories (kg)**[cd]	
Extremely low birth weight (<1)	2 (0.1)
Very low birth weight (1 – < 1.5)	31 (1.2)
Low birth weight (1.5 - <2.5)	578 (22.8)
Normal birth weight (≥2.5)	1,930 (75.9)

[a]Reported for livebirths and stillbirths only
[b]n=50 women had multiple gestations
[c]Reported for livebirths only; missing ultrasound on n=21, hence GA at delivery could not be ascertained
[d]Only reported for those collected within 72 hours; missing data on n=937 infants

For peer review only

There were 98 stillbirths among this cohort, with 75% intrapartum stillbirths (n=74) and 25% antepartum stillbirths (n=24). Nearly three-quarters of neonatal deaths (76.4%, n=139) occurred during the first week of life. The main cause of death during the neonatal period was perinatal asphyxia (39.6%, n=72), preterm birth (19.8%, n=36), infections (12.6%, n=23), and congenital malformations (4.9%, n=9). The remaining causes included other perinatal complications (5.5%, n=10), while for 32 cases (17.6%), the cause could not be ascertained.

In this cohort, the maternal mortality rate is 172 per 100,000 live births, stillbirth rate is 27 per 1000 births, while neonatal mortality rates in the PRISMA Pakistan cohort are 52 per 1,000 live births. The mortality rates for PRISMA study and national mortality rates have been described in table 4. However, since the current study may not have sufficient numbers to comment on maternal mortality, statistical comparison of these estimates was not performed.

Table 4. Rates of maternal and neonatal mortality and stillbirth in PRISMA Pakistan

Indicators	Mortality rates with (95% CI) in the study cohort	Mortality rates in Pakistan (2018-19) (12, 13)
**Maternal mortality rate**	172 (63, 375)/100,000 livebirths	186 /100,000 livebirths
**Stillbirth rate**	27 (22, 33)/1,000 births	44/1,000 births
**Neonatal mortality rate**	52 (45, 60)/1,000 livebirths	51/1,000 livebirths

STRENGTHS AND LIMITATIONS OF THIS STUDY

The prospective nature of the PRISMA Pakistan cohort provides population-based pregnancy outcomes within the specified geographical area in a peri-urban community in Pakistan. This cohort leverages an existing demographic surveillance system to capture pregnancies and follow pregnant women and neonates with low refusal and attrition rates (<10%) as compared to other similar cohorts in the literature (4, 5). The PRISMA study would include 4-5 antenatal touchpoints, labor and delivery information followed by six postnatal touchpoints till 1 year after delivery. These interactions with well-constructed variables at each time point would add considerably rich information regarding pregnancy outcomes in this population. For the current description, we have reported birth and neonatal indicators only, but the main PRISMA study will have infant morbidity and mortality outcomes until one year of life. However, there are certain limitations to this work. Similar to the Global Network Maternal Newborn Health Registry, in our current work, women seek antenatal care in the second trimester

For peer review only - http://bmjopen.bmj.com/site/about/guidelines.xhtml

(5); therefore, our estimates regarding early-pregnancy outcomes, such as miscarriage/abortion, are underestimated given the mean gestational age at enrollment of 17 weeks. However, for the next phase of the PRISMA study, community mobilization efforts will be put in place so that majority of the women enroll in the first trimester of pregnancy. Information on some key variables, such as pregnancy history as well as labor and delivery outcomes for those women who delivered at home and outside the recommended referral facility, is self-reported, thus limiting its interpretation. Another limitation is that not all women from the community are seeking care during pregnancy, and hence, may be different from those enrolled in PRISMA Pakistan, thus limiting the generalizability. This phenomenon has also been reported in other studies where women who do not seek ANC services or seek services later in their pregnancy may be more disadvantaged in terms of their socio-demographic characteristics (14, 15), thus predisposing them to poorer outcomes (16). However, with the existing baseline data acquired through the demographic surveillance system, the study team will be able to compare any differences in health-seeking behaviors of women who did not participate in the study.

COLLABORATION

The study protocol for the next phase of this study, a harmonized multi-site PRISMA open cohort is registered with https://clinicaltrials.gov (number: NCT05904145) or by contacting the corresponding author of this manuscript.

Acknowledgments: The authors acknowledge the contributions of all women who participated in the study.

Contributors:

ZH, FJ, MIN, FF, and ES conceptualized the study and secured funding. ZH, NY, and UK supervised the study at field sites. ZH, SN, AJ, NY, MK, ZHU, and UK were involved in the analysis, interpretation, and writing manuscript. ZH, FJ, MIN, FF, and ES reviewed and provided scientific revision to the manuscript. All authors agreed prior to submission to take responsibility and be accountable for the contents of the manuscript. All authors read and approved the final manuscript.

Funding Statement: This study is funded by the Bill & Melinda Gates Foundation (INV-005776**).**

Competing Interests: None declared

For peer review only - http://bmjopen.bmj.com/site/about/guidelines.xhtml

References:

1. Manolio TA, Bailey-Wilson JE, Collins FS. Genes, environment and the value of prospective cohort studies. Nat Rev Genet. 2006;7(10):812-20.
2. Lawlor DA, Andersen AM, Batty GD. Birth cohort studies: past, present and future. Int J Epidemiol. 2009;38(4):897-902.
3. Baqui A, Ahmed P, Dasgupta SK, Begum N, Rahman M, Islam N, et al. Development and validation of a simplified algorithm for neonatal gestational age assessment - protocol for the Alliance for Maternal Newborn Health Improvement (AMANHI) prospective cohort study. J Glob Health. 2017;7(2):021201.
4. Patel KK, Rai R, Rai AK. Determinants of infant mortality in Pakistan: evidence from Pakistan Demographic and Health Survey 2017–18. Journal of Public Health. 2021;29:693-701.
5. McClure EM, Garces AL, Hibberd PL, Moore JL, Goudar SS, Saleem S, et al. The Global Network Maternal Newborn Health Registry: a multi-country, community-based registry of pregnancy outcomes. Reprod Health. 2020;17(Suppl 2):184.
6. Ilyas M, Naeem K, Fatima U, Nisar MI, Kazi AM, Jehan F, et al. Profile: health and demographic surveillance system in peri-urban areas of Karachi, Pakistan. Gates Open Research. 2018.
7. GE Vivid iq [Available from: https://www.probomedical.com/shop/ultrasound-systems/ge/ge-vivid-iq/.
8. Organization WH. WHO recommendations on antenatal care for a positive pregnancy experience: World Health Organization; 2016.
9. Nichols EK, Byass P, Chandramohan D, Clark SJ, Flaxman AD, Jakob R, et al. The WHO 2016 verbal autopsy instrument: An international standard suitable for automated analysis by InterVA, InSilicoVA, and Tariff 2.0. PLoS medicine. 2018;15(1):e1002486.
10. D'Agostino Sr RB, Vasan RS, Pencina MJ, Wolf PA, Cobain M, Massaro JM, et al. General cardiovascular risk profile for use in primary care: the Framingham Heart Study. Circulation. 2008;117(6):743-53.
11. Momjian B. PostgreSQL Introduction and Concepts Bruce Momjian ADDISON. 2001.
12. Nousheen I, Safia A, Urooj R, Muhammad Waqas K, Sohail B, Mariam F, et al. Difference in gender perception regarding smokeless tobacco consumption; A cross sectional survey from Karachi Pakistan. European Respiratory Journal. 2019;54(suppl 63):PA706.
13. Aziz A, Saleem S, Nolen TL, Pradhan NA, McClure EM, Jessani S, et al. Why are the Pakistani maternal, fetal and newborn outcomes so poor compared to other low and middle-income countries? Reprod Health. 2020;17(Suppl 3):190.
14. Nisar YB, Aurangzeb B, Dibley MJ, Alam A. Qualitative exploration of facilitating factors and barriers to use of antenatal care services by pregnant women in urban and rural settings in Pakistan. BMC pregnancy and childbirth. 2016;16:1-9.
15. Agha S, Tappis H. The timing of antenatal care initiation and the content of care in Sindh, Pakistan. BMC pregnancy and childbirth. 2016;16:1-9.
16. Alwan NA, Roderick PJ, Macklon NS. Is timing of the first antenatal visit associated with adverse birth outcomes? Analysis from a population-based birth cohort. The Lancet. 2016;388:S18.

For peer review only

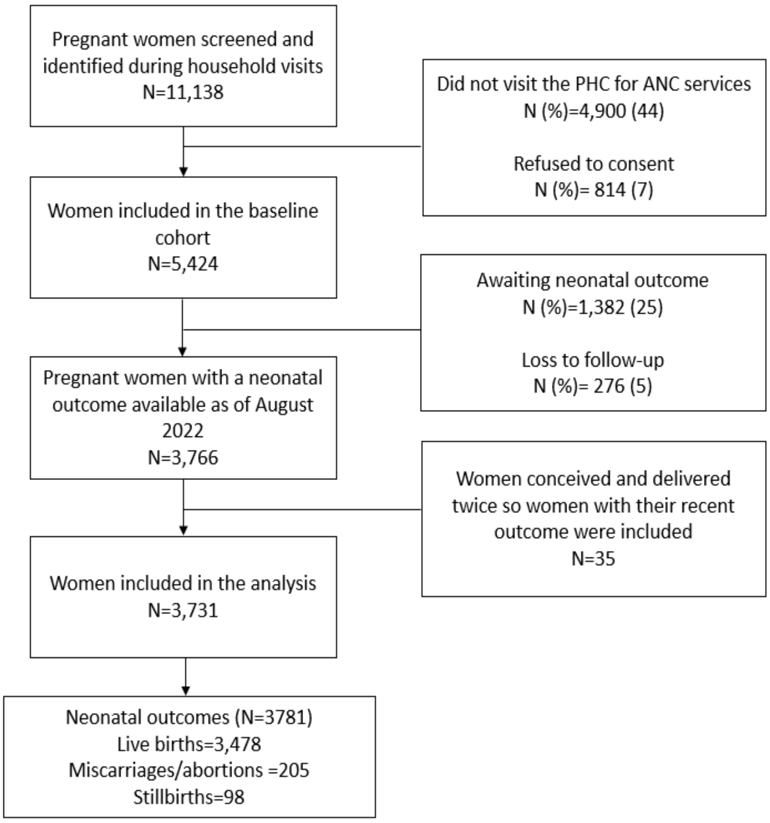

PHC= Primary healthcare, ANC= Antenatal care

Supplemental Figure 1. PRISMA study sites in Karachi, Pakistan

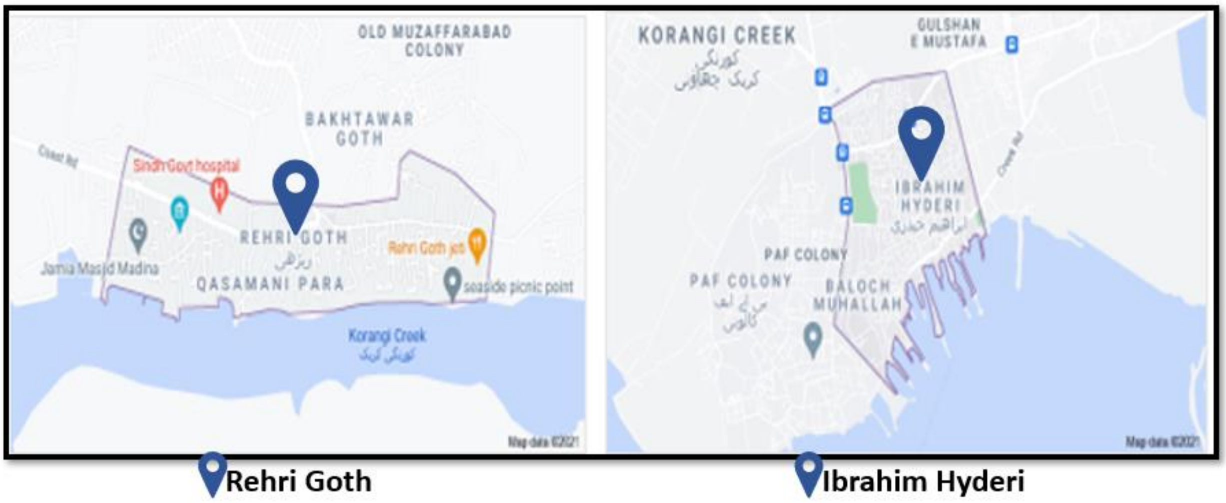

For peer review only

STROBE Statement—Checklist of items that should be included in reports of ***cohort studies***

	Item No	Recommendation	Page No
**Title and abstract**	1	(*a*) Indicate the study's design with a commonly used term in the title or the abstract	1
		(*b*) Provide in the abstract an informative and balanced summary of what was done and what was found	2-3
**Introduction**			
Background/rationale	2	Explain the scientific background and rationale for the investigation being reported	5
Objectives	3	State specific objectives, including any prespecified hypotheses	5
**Methods**			
Study design	4	Present key elements of study design early in the paper	6
Setting	5	Describe the setting, locations, and relevant dates, including periods of recruitment, exposure, follow-up, and data collection	6
Participants	6	(*a*) Give the eligibility criteria, and the sources and methods of selection of participants. Describe methods of follow-up	6
		(*b*) For matched studies, give matching criteria and number of exposed and unexposed	NA
Variables	7	Clearly define all outcomes, exposures, predictors, potential confounders, and effect modifiers. Give diagnostic criteria, if applicable	NA
Data sources/ measurement	8*	For each variable of interest, give sources of data and details of methods of assessment (measurement). Describe comparability of assessment methods if there is more than one group	7-9
Bias	9	Describe any efforts to address potential sources of bias	NA
Study size	10	Explain how the study size was arrived at	NA
Quantitative variables	11	Explain how quantitative variables were handled in the analyses. If applicable, describe which groupings were chosen and why	NA
Statistical methods	12	(*a*) Describe all statistical methods, including those used to control for confounding	NA
		(*b*) Describe any methods used to examine subgroups and interactions	NA
		(*c*) Explain how missing data were addressed	NA
		(*d*) If applicable, explain how loss to follow-up was addressed	NA
		(*e*) Describe any sensitivity analyses	NA
**Results**			
Participants	13*	(a) Report numbers of individuals at each stage of study—eg numbers potentially eligible, examined for eligibility, confirmed eligible, included in the study, completing follow-up, and analysed	6
		(b) Give reasons for non-participation at each stage	NA
		(c) Consider use of a flow diagram	6
Descriptive data	14*	(a) Give characteristics of study participants (eg demographic, clinical, social) and information on exposures and potential confounders	7
		(b) Indicate number of participants with missing data for each variable of interest	NA
		(c) Summarise follow-up time (eg, average and total amount)	8-9
Outcome data	15*	Report numbers of outcome events or summary measures over time	NA

For peer review only

For peer review only - http://bmjopen.bmj.com/site/about/guidelines.xhtml

Main results	16	(*a*) Give unadjusted estimates and, if applicable, confounder-adjusted estimates and their precision (eg, 95% confidence interval). Make clear which confounders were adjusted for and why they were included	NA
		(*b*) Report category boundaries when continuous variables were categorized	NA
		(*c*) If relevant, consider translating estimates of relative risk into absolute risk for a meaningful time period	NA
Other analyses	17	Report other analyses done—eg analyses of subgroups and interactions, and sensitivity analyses	NA
**Discussion**			
Key results	18	Summarise key results with reference to study objectives	9-12
Limitations	19	Discuss limitations of the study, taking into account sources of potential bias or imprecision. Discuss both direction and magnitude of any potential bias	12-13
Interpretation	20	Give a cautious overall interpretation of results considering objectives, limitations, multiplicity of analyses, results from similar studies, and other relevant evidence	9-12
Generalisability	21	Discuss the generalisability (external validity) of the study results	12
**Other information**			
Funding	22	Give the source of funding and the role of the funders for the present study and, if applicable, for the original study on which the present article is based	13

*Give information separately for exposed and unexposed groups.

Note: An Explanation and Elaboration article discusses each checklist item and gives methodological background and published examples of transparent reporting. The STROBE checklist is best used in conjunction with this article (freely available on the Web sites of PLoS Medicine at http://www.plosmedicine.org/, Annals of Internal Medicine at http://www.annals.org/, and Epidemiology at http://www.epidem.com/). Information on the STROBE Initiative is available at http://www.strobe-statement.org.

For peer review only

BMJ Open

Cohort profile: The Pregnancy Risk Infant Surveillance and Measurement Alliance (PRISMA) - Pakistan

Journal:	*BMJ Open*
Manuscript ID	bmjopen-2023-078222.R3
Article Type:	Cohort profile
Date Submitted by the Author:	22-Nov-2023

Complete List of Authors:	Naz, Sabahat; The Aga Khan University, Department of Pediatrics and Child Health<br>Jaffar, Ali; The Aga Khan University, Department of Pediatrics and Child Health<br>Yazdani, Nida; VITAL Pakistan<br>Kashif, Muhammad ; The Aga Khan University, Department of Pediatrics and Child Health<br>Hussain, Zaid; The Aga Khan University, Department of Pediatrics and Child Health<br>Khan, Uzma; VITAL Pakistan<br>Farooq, Fouzia; The George Washington University, Department of Global Health<br>Nisar, Muhammad; The Aga Khan University, Department of Pediatrics and Child Health<br>Jehan, Fyezah; The Aga Khan University, Department of Pediatrics and Child Health<br>Smith, Emily ; The George Washington University, Public Health<br>Hoodbhoy, Zahra; The Aga Khan University, Department of Pediatrics and Child Health

**Primary Subject Heading**:	Public health
Secondary Subject Heading:	Epidemiology, Paediatrics, Public health
Keywords:	Follow-Up Studies, Community child health < PAEDIATRICS, Primary Health Care, PUBLIC HEALTH, Risk Factors

SCHOLARONE™ Manuscripts

I, the Submitting Author has the right to grant and does grant on behalf of all authors of the Work (as defined in the below author licence), an exclusive licence and/or a non-exclusive licence for contributions from authors who are: i) UK Crown employees; ii) where BMJ has agreed a CC-BY licence shall apply, and/or iii) in accordance with the terms applicable for US Federal Government officers or employees acting as part of their official duties; on a worldwide, perpetual, irrevocable, royalty-free basis to BMJ Publishing Group Ltd ("BMJ") its licensees and where the relevant Journal is co-owned by BMJ to the co-owners of the Journal, to publish the Work in this journal and any other BMJ products and to exploit all rights, as set out in our licence.

The Submitting Author accepts and understands that any supply made under these terms is made by BMJ to the Submitting Author unless you are acting as an employee on behalf of your employer or a postgraduate student of an affiliated institution which is paying any applicable article publishing charge ("APC") for Open Access articles. Where the Submitting Author wishes to make the Work available on an Open Access basis (and intends to pay the relevant APC), the terms of reuse of such Open Access shall be governed by a Creative Commons licence – details of these licences and which Creative Commons licence will apply to this Work are set out in our licence referred to above.

Other than as permitted in any relevant BMJ Author's Self Archiving Policies, I confirm this Work has not been accepted for publication elsewhere, is not being considered for publication elsewhere and does not duplicate material already published. I confirm all authors consent to publication of this Work and authorise the granting of this licence.

For peer review only

Title: Cohort profile: The Pregnancy Risk Infant Surveillance and Measurement Alliance (PRISMA) - Pakistan

Authors: Sabahat Naz[1], Ali Jaffar[1], Nida Yazdani[2], Muhammad Kashif[1], Zaid Hussain[1], Uzma Khan[2], Fouzia Farooq[3], Muhammad Imran Nisar[1], Fyezah Jehan[1], Emily R. Smith[3], Zahra Hoodbhoy[1]

[1]Department of Pediatrics and Child Health, The Aga Khan University, Stadium Road, Karachi, Pakistan 74800

[2]VITAL Pakistan Trust, Karachi, Pakistan

[3]Department of Global Health, Milken Institute School of Public Health, The George Washington University, Washington, D.C. 20052, USA

Corresponding author:

Zahra Hoodbhoy

Department of Pediatrics and Child Health

The Aga Khan University

Stadium Road, Karachi, Pakistan 74800

Tel No: 92-2134864126

Email: zahra.hoodbhoy@aku.edu

For peer review only

Abstract

Purpose:

Pakistan has disproportionately high maternal and neonatal morbidity and mortality. There is a lack of detailed, population-representative data to provide evidence for risk factors, morbidities, and mortality among pregnant women and their newborns. The Pregnancy Risk, Infant Surveillance, and Measurement Alliance (PRISMA) is a multi-country open cohort that aims to collect high-dimensional, standardized data across five South Asian and African countries for estimating risk and developing innovative strategies to optimize pregnancy outcomes for mothers and their newborns. This study presents the baseline maternal and neonatal characteristics of the Pakistan site occurring prior to the launch of a multi-site, harmonized protocol.

Participants:

PRISMA Pakistan study is being conducted at two peri-urban field sites in Karachi, Pakistan. These sites have primary health care clinics where pregnant women and their newborns are followed during the antenatal, intrapartum, and postnatal periods up to one year after delivery. All encounters are captured electronically through a custom-built Android application. A total of 3,731 pregnant women with a mean age of 26.6 ± 5.8 years at the time of pregnancy with neonatal outcomes between January 2021 and August 2022 serve as a baseline for the PRISMA Pakistan study.

Findings to date:

In this cohort, live births accounted for the majority of pregnancy outcomes (92%, n=3,478), followed by miscarriages/abortions (5.5%, n=205) and stillbirths (2.6%, n=98). Twenty-two percent of women (n=786) delivered at home. One out of every four neonates was low birth weight

For peer review only - http://bmjopen.bmj.com/site/about/guidelines.xhtml

(< 2500 grams), and one out of every five was preterm (gestational age <37 weeks). The maternal mortality rate was 172/100,000 pregnancies, the neonatal mortality rate was 52/1000 live births, and the stillbirth rate was 27/1000 births. The three most common causes of neonatal deaths obtained through verbal autopsy were perinatal asphyxia (39.6%), preterm births (19.8%), and infections (12.6%).

Future plans:

The PRISMA cohort will provide data-driven insights to prioritize and design interventions to improve maternal and neonatal outcomes in low-resource regions.

Trial registration number: NCT05904145

For peer review only

Strengths and limitations of this study

- The prospective nature of the PRISMA Pakistan cohort enables the collection of high-quality data from attendees of the PHC available in the community within the defined geographical area.
- This is a unique longitudinal cohort of attendees of the PHC available in the community in Pakistan with the ability to leverage an existing demographic surveillance system to capture early pregnancies and follow these women postpartum with low attrition rates.
- Although women are encouraged to deliver at the recommended referral facility for obtaining their information, nearly 36% delivered at other health facilities or their homes. In such cases, data on key labor and delivery outcomes is self-reported, thus limiting its interpretation.

For peer review only

For peer review only - http://bmjopen.bmj.com/site/about/guidelines.xhtml

INTRODUCTION:

Population-based longitudinal birth cohorts provide important data to study a life course approach to in-utero exposures and etiological factors that contribute to developing diseases early and later in life (1). The advantages of such cohort studies include standardized data collection, a systematic approach in following participants throughout the study, and the generalizability of findings to the larger population (1). In addition, it allows investigators to collect accurate information on the exposures, outcomes, potential confounders, and other important characteristics at the individual as well as population level (2). Early identification of these risk factors may provide an opportunity to design and implement population-based preventive strategies to optimize individual and population-level outcomes (3).

Establishing robust perinatal datasets in low-resource settings is essential for tracking local demographic shifts related to risk factors, morbidities and mortality outcomes among pregnant women and their newborns (4). This information can also aid in the development of stratified care pathways for efficient resource utilization, thus improving outcomes (3). The Demographic Health Survey (DHS) and the Maternal Newborn Health Registry of the NICHD Global Network for Women's and Children's Health Research are examples that have generated a wealth of data over a decade in low and middle-income (LMICs) countries, including Pakistan (4, 5). However, these datasets are either cross-sectional (i.e., DHS) or have limited timepoints during the pregnancy care continuum. Furthermore, these datasets focus on maternal and neonatal mortality as primary outcomes and lack detailed clinical and laboratory data on major morbidities in the mother and infant.

To address these gaps, the Pregnancy Risk Infant Surveillance and Measurement Alliance (PRISMA) Maternal and Newborn Health (MNH) study is being conducted across five sites in South Asia (India, Pakistan) and Sub-Saharan Africa (Zambia, Kenya, Ghana) to longitudinally assess pregnancy risk factors and their relationships with poor pregnancy outcomes, such as stillbirth, neonatal morbidity and mortality, and maternal mortality and severe maternal and infant morbidity. This multi-country, open cohort study aims to provide a harmonized dataset to quantify the burden of maternal, fetal, and neonatal risk factors and understand vulnerabilities, morbidity, and mortality.

The objective of this paper was to provide an overview of the baseline characteristics of women in the study catchment area for the Pakistan site before the initiation of the PRISMA study.

For peer review only - http://bmjopen.bmj.com/site/about/guidelines.xhtml

COHORT DESCRIPTION:

Study design and participants

In Pakistan, the PRISMA study is being conducted in two peri-urban community settings in Karachi, namely Rehri Goth and Ibrahim Hyderi; the areas comprise approximately 23,170 households and a population of 227,549 individuals (refer to Supplemental Figure 1).

The existing maternal and child health surveillance system at these sites will be leveraged for the early identification of pregnancies for the PRISMA cohort. The Department of Pediatrics and Child Health at the Aga Khan University (AKU) has been conducting maternal and child surveillance at the peri-urban coastal field sites since 2003 (6). This system gathers data on vital events regarding married women of reproductive age and children under five years of age. Records of births, deaths, pregnancies, and migration events are documented through quarterly household visits by Community Health Workers (CHWs) using the existing household rosters of these sites.

Pregnant women captured through pregnancy surveillance are encouraged to visit the primary health care (PHC) clinics located within the community for antenatal care. These PHCs are run by AKU and are not part of the public health system. Antenatal care, along with laboratory and ultrasound procedures, is offered free of cost for all pregnant women from the catchment area by community midwives located at these centers. Pregnant women aged 15 - 49 years with a confirmed intrauterine pregnancy of gestational age (GA) <20 weeks via standard ultrasound (GE Vivid IQ) (7) are eligible to participate in the study. A total of 3,731 pregnant women with a neonatal outcome of 3781 between January 2021 to August 2022 serve as a baseline for PRISMA Pakistan. The flow chart of this population is described in Figure 1.

Figure 1. Flow diagram for PRISMA enrollment status January 2021 to August 2022

Measures and procedure

At baseline, pregnant women visit the PHC located within the study catchment area for antenatal care services. At the first antenatal care (ANC) visit, the pregnancy history of the women along with anthropometry, blood pressure and other vital information regarding the current pregnancy is obtained. The pregnant woman has her ultrasound to establish the GA at the first visit followed by laboratory investigations (including hemoglobin, hepatitis B and C screening). The subsequent ANC visit dates are given as per WHO recommendations for ANC visits (8). During delivery, women are provided transport to our partnering hospital to ensure skilled labor and delivery. In cases where women give birth at home or at another facility

For peer review only

besides our partnering facility, community health workers (CHWs) visit the household to obtain information regarding labor and delivery, complications, if any, and perform newborn measurements (weight, length, and head circumference) within 72 hours of life. In case of a maternal or neonatal mortality, a verbal autopsy is performed using the WHO 2016 verbal autopsy instrument (9) by trained research staff and coded by a physician to ascertain cause of death.

All visits are captured electronically through a custom-built Android application. This application uses an Open Smart Register Platform, an open-source mobile health platform designed to support the WHO SMART guidelines for data-driven decision-making in healthcare (10). Once entered into the application, data is extracted, transformed, and loaded into a PostgreSQL (11) relational database management system to allow for monitoring, reporting, and analytics. The application is user-specific and team-based (midwives, CHWs), with each team having a unique ID and password for data collection. The coordinators at the study sites perform period data checks and monitoring in order to ensure accurate data collection.

Patient and public involvement:

Written informed consent is obtained from all participants in the local language (Urdu). For illiterate participants, consent was obtained through a trusted witness who explained the consent form in the participant's language. A thumbprint or mark was taken from the participant, with both the participant and the witness signing the form to validate the process. The cohort participants are not involved in the design or recruitment of the study. However, the community mobilization teams of PRISMA Pakistan have been actively engaged with community stakeholders and pregnant women to inform on the study and benefits of early initiation of antenatal care.

Ethical Approval:

The PRISMA MNH Pakistan baseline study received ethical approval in Pakistan (001-VPT-IRB-20).

FINDINGS TO DATE:

The sociodemographic and clinical characteristics of women included, as well as those excluded from the study, are described in Table 1.

For peer review only

Table 1. Sociodemographic and clinical characteristics of women included and excluded in the baseline cohort of PRISMA Pakistan

Characteristics	Women included in the analysis (n = 3,731) Mean ± SD, Median (range) or n (%)	Women who did not receive ANC services or did not consent (n= 5,714) Mean ± SD, Median (range) or n (%)
Age at marriage (years)	20.1 ± 3.2	28.2 ± 6.1
**Maternal education**		
Literate	1,198 (33.1)	1,934 (38.4)
**Women's employment status**		
Employed	1,129 (31.2)	-
**Husband's employment status**		
Employed	3,582 (98.9)	-
**Wealth Index (quintiles)**		-
Q1 (least wealthy)	791 (21.8)	
Q2	659 (18.2)	
Q3	736 (20.3)	
Q4	813 (22.4)	
Q5 (wealthiest)	624 (17.2)	
Reported Smoking	95 (2.6)	-
Current use of smokeless tobacco	818 (21.9)	-
Total number of pregnancies	3 (1 - 16)	3 (1 – 20)
Primigravida	836 (22.4)	826 (16.3)
Multigravida (2 – 5)	2,232 (59.8)	2,576 (51)
Grand Multigravida (>5)	663 (17.8)	1,654 (32.7)
No. of previous livebirths	2 (1 - 14)	3 (1 – 15)
No. of previous stillbirths	1 (1 - 5)	1 (1 – 9)
No. of previous miscarriages	1 (1 - 10)	1 (1 – 9)
No. of previous induced abortions	1 (1 - 3)	1 (1 – 5)

For peer review only - http://bmjopen.bmj.com/site/about/guidelines.xhtml

As of August 2022, 3,731 women with pregnancy outcomes serve as a baseline for the PRISMA Pakistan cohort. Women enrolled in this cohort have a mean age of 26.6 ± 5.8 years at the time of pregnancy. The average number of antenatal visits were 5.4 ± 2.8, with a mean GA of 17.2 ± 8.1 weeks at the first visit. Using the cutoff of mid-upper arm circumference (MUAC) of <23 cm, 27% (n=1,006) of pregnant were malnourished. Approximately two-thirds of women (64%, n=2570) had either mild, moderate, or severe anemia. Detailed pregnancy characteristics of these women are described in Table 2.

Table 2. Pregnancy characteristics of enrolled participants

Characteristics	Total Population (n = 3,731) Mean ± SD; n (%)
Age at the time of pregnancy (years)	26.6 ± 5.8
Number of ANC visits	5.4 ± 2.8
GA at first ANC visit (weeks)	17.2 ± 8.1
MUAC* (cm)	25.8 ± 4.1
**Nutritional status based on MUAC (cm)**	
Normal (≥23)	2,724 (73.0)
Malnourished (<23)	1,006 (27.0)
BMI (kg/m^2)	23.4 ± 5.2
**Nutritional status based on BMI (kg/m^2)**	
Underweight (<18.5)	564 (15.1)
Normal weight (18.5 – 22.9)	1,454 (39.0)
Overweight (23.0 – <26.9)	891 (23.9)
Obese (≥27.0)	821 (22.0)
Hemoglobin levels (g/dL)	10.3 ± 1.4
**Anemia status (hemoglobin level)**	
Normal (≥11.0 g/dL)	1,161 (35.7)
Mild Anemia (10.0 - 10.9 g/dL)	940 (28.9)
Moderate Anemia (7.0 - 9.9 g/dL)	1,077 (33.1)
Severe Anemia (<7.0 g/dL)	71 (2.2)
Chronic Hypertension	124 (3.3)
Diabetes Mellitus	23 (0.6)
Gestational Hypertension	48 (1.3)
Hepatitis B	33 (1.2)
Hepatitis C	52 (1.9)

BMI, Body mass index; GA, Gestational age; MUAC, Mid-upper arm circumference

For peer review only - http://bmjopen.bmj.com/site/about/guidelines.xhtml

Majority of the pregnancy outcomes in this cohort were live births (92%, n=3,478) followed by miscarriages/abortions (5.5%, n=205) and stillbirths (2.6%, n=98). Twenty-two percent (n=786) of these women delivered at home. Overall, 20.1% (n=696) infants were preterm, and 24.1% (n=611) were low birthweight (Table 3).

Table 3. Delivery characteristics and neonatal outcomes in the study population

Characteristics	n (%)
**Number of Pregnant Women**	**3,731**
**Number of fetuses**	
Single	3,681 (98.7)
Multiple gestation	50 (1.3)
**Place of delivery**[a]	
Partner referral facility	2,288 (64.0)
Other facilities	502 (14.0)
Home births	786 (22.0)
**Mode of delivery**[a]	
Vaginal	2,620 (73.3)
Spontaneous	2617 (99.9)
Assisted	3 (0.1)
Cesarean	956 (26.7)
Elective	530 (55.7)
Emergent	422 (44.3)
**Number of Birth Outcomes**	**3781**
**Birth outcomes**[b]	
Livebirths	3,478 (92.0)
Stillbirths	98 (2.6)
Miscarriages	180 (4.8)
Abortions	25 (0.7)
**Gender**[a]	
Male	1,836 (51.3)
Female	1,738 (48.6)
Ambiguous	2 (0.0)
**Gestational age at delivery (weeks)**[c]	
Extremely preterm (<28)	8 (0.2)
Very preterm (28 - <32)	44 (1.3)
Moderate-to-late preterm (32 - < 37)	644 (18.6)
Term (≥37)	2,761 (79.9)
**Birth weight categories (kg)**[cd]	
Extremely low birth weight (<1)	2 (0.1)
Very low birth weight (1 – < 1.5)	31 (1.2)
Low birth weight (1.5 - <2.5)	578 (22.8)
Normal birth weight (≥2.5)	1,930 (75.9)

[a]Reported for livebirths and stillbirths only
[b]n=50 women had multiple gestations
[c]Reported for livebirths only; missing ultrasound on n=21, hence GA at delivery could not be ascertained
[d]Only reported for those collected within 72 hours; missing data on n=937 infants

For peer review only

There were 98 stillbirths among this cohort, with 75% intrapartum stillbirths (n=74) and 25% antepartum stillbirths (n=24). Nearly three-quarters of neonatal deaths (76.4%, n=139) occurred during the first week of life. The main cause of death during the neonatal period was perinatal asphyxia (39.6%, n=72), preterm birth (19.8%, n=36), infections (12.6%, n=23), and congenital malformations (4.9%, n=9). The remaining causes included other perinatal complications (5.5%, n=10), while for 32 cases (17.6%), the cause could not be ascertained.

In this cohort, the maternal mortality rate is 172 per 100,000 live births, stillbirth rate is 27 per 1000 births, while neonatal mortality rates in the PRISMA Pakistan cohort are 52 per 1,000 live births. The mortality rates for PRISMA study and national mortality rates (12, 13) have been described in Table 4. However, since the current study may not have sufficient numbers to comment on maternal mortality, statistical comparison of these estimates was not performed.

Table 4. Rates of maternal and neonatal mortality and stillbirth in PRISMA Pakistan

Indicators	Mortality rates with (95% CI) in the study cohort	Mortality rates in Pakistan (2018-19)
**Maternal mortality rate**	172 (63, 375)/100,000 livebirths	186 /100,000 livebirths
**Stillbirth rate**	27 (22, 33)/1,000 births	44/1,000 births
**Neonatal mortality rate**	52 (45, 60)/1,000 livebirths	51/1,000 livebirths

STRENGTHS AND LIMITATIONS OF THIS STUDY

The prospective nature of the PRISMA Pakistan cohort provides pregnancy outcomes from the attendees of the PHC available in the community within the specified geographical area in a peri-urban community in Pakistan. This cohort leverages an existing demographic surveillance system to capture pregnancies and follow pregnant women and neonates with low refusal and attrition rates (<10%) as compared to other similar cohorts in the literature (4, 5). The PRISMA study would include 4-5 antenatal touchpoints, labor and delivery information followed by six postnatal touchpoints till 1 year after delivery. These interactions with well-constructed variables at each time point would add considerably rich information regarding pregnancy outcomes in this population. For the current description, we have reported birth and neonatal indicators only, but the main PRISMA study will have infant morbidity and mortality outcomes until one year of life. However, there are certain limitations to this work. Similar to the Global Network Maternal Newborn Health Registry, in our current work,

For peer review only - http://bmjopen.bmj.com/site/about/guidelines.xhtml

women seek antenatal care in the second trimester (5); therefore, our estimates regarding early-pregnancy outcomes, such as miscarriage/abortion, are underestimated given the mean gestational age at enrollment of 17 weeks. However, for the next phase of the PRISMA study, community mobilization efforts will be put in place so that majority of the women enroll in the first trimester of pregnancy. Information on some key variables, such as pregnancy history as well as labor and delivery outcomes for those women who delivered at home and outside the recommended referral facility, is self-reported, thus limiting its interpretation. Another limitation is that not all women from the community are seeking care during pregnancy, and hence, may be different from those enrolled in PRISMA Pakistan, thus limiting the generalizability. This phenomenon has also been reported in other studies where women who do not seek ANC services or seek services later in their pregnancy may be more disadvantaged in terms of their socio-demographic characteristics (14, 15), thus predisposing them to poorer outcomes (16). However, with the existing baseline data acquired through the demographic surveillance system, the study team will be able to compare any differences in health-seeking behaviors of women who did not participate in the study.

For peer review only

COLLABORATION

The study protocol for the next phase of this study, a harmonized multi-site PRISMA open cohort is registered with https://clinicaltrials.gov (number: NCT05904145) or by contacting the corresponding author of this manuscript.

Acknowledgments: The authors acknowledge the contributions of all women who participated in the study.

Contributors:

ZH, FJ, MIN, FF, and ES conceptualized the study and secured funding. ZH, NY, and UK supervised the study at field sites. ZH, SN, AJ, NY, MK, ZHU, and UK were involved in the analysis, interpretation, and writing manuscript. ZH, FJ, MIN, FF, and ES reviewed and provided scientific revision to the manuscript. All authors agreed prior to submission to take responsibility and be accountable for the contents of the manuscript. All authors read and approved the final manuscript.

Funding Statement: This study is funded by the Bill & Melinda Gates Foundation (INV-005776**).**

Competing Interests: None declared

For peer review only - http://bmjopen.bmj.com/site/about/guidelines.xhtml

References:

1. Manolio TA, Bailey-Wilson JE, Collins FS. Genes, environment and the value of prospective cohort studies. Nat Rev Genet. 2006;7(10):812-20.
2. Lawlor DA, Andersen AM, Batty GD. Birth cohort studies: past, present and future. Int J Epidemiol. 2009;38(4):897-902.
3. Baqui A, Ahmed P, Dasgupta SK, Begum N, Rahman M, Islam N, et al. Development and validation of a simplified algorithm for neonatal gestational age assessment - protocol for the Alliance for Maternal Newborn Health Improvement (AMANHI) prospective cohort study. J Glob Health. 2017;7(2):021201.
4. Patel KK, Rai R, Rai AK. Determinants of infant mortality in Pakistan: evidence from Pakistan Demographic and Health Survey 2017–18. Journal of Public Health. 2021;29:693-701.
5. McClure EM, Garces AL, Hibberd PL, Moore JL, Goudar SS, Saleem S, et al. The Global Network Maternal Newborn Health Registry: a multi-country, community-based registry of pregnancy outcomes. Reprod Health. 2020;17(Suppl 2):184.
6. Ilyas M, Naeem K, Fatima U, Nisar MI, Kazi AM, Jehan F, et al. Profile: health and demographic surveillance system in peri-urban areas of Karachi, Pakistan. Gates Open Research. 2018.
7. GE Vivid iq [Available from: https://www.probomedical.com/shop/ultrasound-systems/ge/ge-vivid-iq/.
8. Organization WH. WHO recommendations on antenatal care for a positive pregnancy experience: World Health Organization; 2016.
9. Nichols EK, Byass P, Chandramohan D, Clark SJ, Flaxman AD, Jakob R, et al. The WHO 2016 verbal autopsy instrument: An international standard suitable for automated analysis by InterVA, InSilicoVA, and Tariff 2.0. PLoS medicine. 2018;15(1):e1002486.
10. [Available from: https://smartregister.org/#:~:text=OpenSRP%20is%20a%20%E2%80%9Cnext%20gen,systems%20like%20DHIS2%20or%20OpenMRS.
11. Momjian B. PostgreSQL Introduction and Concepts Bruce Momjian ADDISON. 2001.
12. National Institute of Population Studies (NIPS) [Pakistan] and ICF. 2020. Pakistan Maternal Mortality Survey 2019: Key Indicators Report. Islamabad, Pakistan, and Rockville, Maryland, USA: NIPS and ICF.
13. Aziz A, Saleem S, Nolen TL, Pradhan NA, McClure EM, Jessani S, et al. Why are the Pakistani maternal, fetal and newborn outcomes so poor compared to other low and middle-income countries? Reprod Health. 2020;17(Suppl 3):190.
14. Nisar YB, Aurangzeb B, Dibley MJ, Alam A. Qualitative exploration of facilitating factors and barriers to use of antenatal care services by pregnant women in urban and rural settings in Pakistan. BMC pregnancy and childbirth. 2016;16:1-9.
15. Agha S, Tappis H. The timing of antenatal care initiation and the content of care in Sindh, Pakistan. BMC pregnancy and childbirth. 2016;16:1-9.
16. Alwan NA, Roderick PJ, Macklon NS. Is timing of the first antenatal visit associated with adverse birth outcomes? Analysis from a population-based birth cohort. The Lancet. 2016;388:S18.

For peer review only - http://bmjopen.bmj.com/site/about/guidelines.xhtml

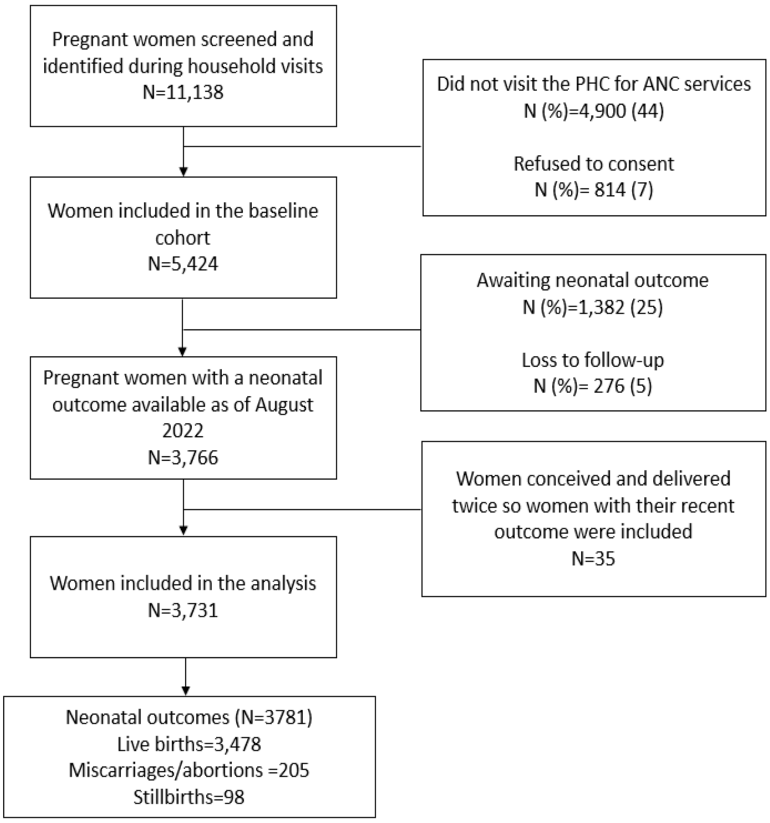

PHC= Primary healthcare, ANC= Antenatal care

review only

Supplemental Figure 1. PRISMA study sites in Karachi, Pakistan

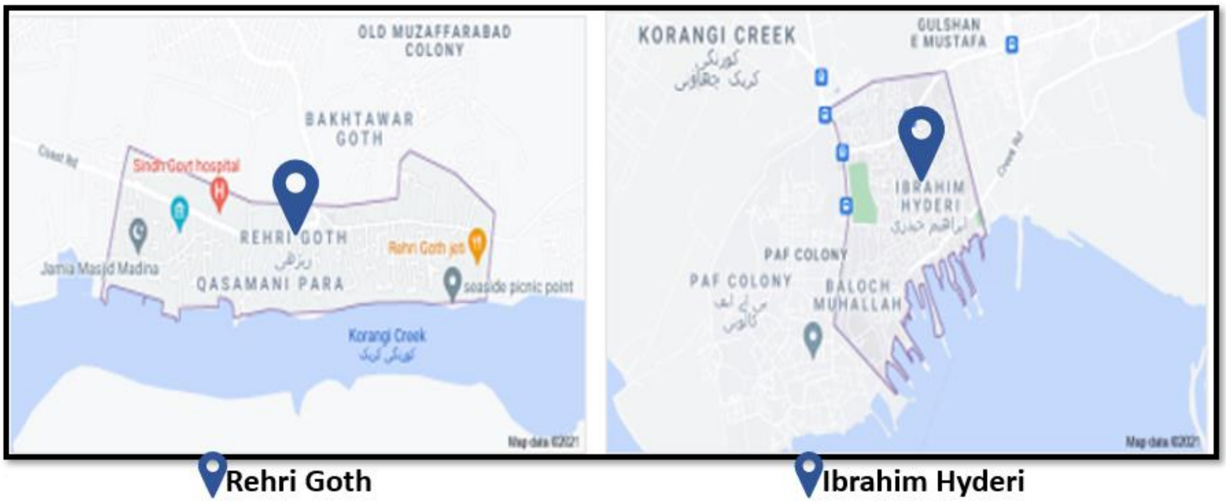

For peer review only

STROBE Statement—Checklist of items that should be included in reports of ***cohort studies***

	Item No	Recommendation	Page No
**Title and abstract**	1	(*a*) Indicate the study's design with a commonly used term in the title or the abstract	1
		(*b*) Provide in the abstract an informative and balanced summary of what was done and what was found	2-3
**Introduction**			
Background/rationale	2	Explain the scientific background and rationale for the investigation being reported	5
Objectives	3	State specific objectives, including any prespecified hypotheses	5
**Methods**			
Study design	4	Present key elements of study design early in the paper	6
Setting	5	Describe the setting, locations, and relevant dates, including periods of recruitment, exposure, follow-up, and data collection	6
Participants	6	(*a*) Give the eligibility criteria, and the sources and methods of selection of participants. Describe methods of follow-up	6
		(*b*) For matched studies, give matching criteria and number of exposed and unexposed	NA
Variables	7	Clearly define all outcomes, exposures, predictors, potential confounders, and effect modifiers. Give diagnostic criteria, if applicable	NA
Data sources/ measurement	8*	For each variable of interest, give sources of data and details of methods of assessment (measurement). Describe comparability of assessment methods if there is more than one group	7-9
Bias	9	Describe any efforts to address potential sources of bias	NA
Study size	10	Explain how the study size was arrived at	NA
Quantitative variables	11	Explain how quantitative variables were handled in the analyses. If applicable, describe which groupings were chosen and why	NA
Statistical methods	12	(*a*) Describe all statistical methods, including those used to control for confounding	NA
		(*b*) Describe any methods used to examine subgroups and interactions	NA
		(*c*) Explain how missing data were addressed	NA
		(*d*) If applicable, explain how loss to follow-up was addressed	NA
		(*e*) Describe any sensitivity analyses	NA
**Results**			
Participants	13*	(a) Report numbers of individuals at each stage of study—eg numbers potentially eligible, examined for eligibility, confirmed eligible, included in the study, completing follow-up, and analysed	6
		(b) Give reasons for non-participation at each stage	NA
		(c) Consider use of a flow diagram	6
Descriptive data	14*	(a) Give characteristics of study participants (eg demographic, clinical, social) and information on exposures and potential confounders	7
		(b) Indicate number of participants with missing data for each variable of interest	NA
		(c) Summarise follow-up time (eg, average and total amount)	8-9
Outcome data	15*	Report numbers of outcome events or summary measures over time	NA

For peer review only

For peer review only - http://bmjopen.bmj.com/site/about/guidelines.xhtml

Main results	16	(*a*) Give unadjusted estimates and, if applicable, confounder-adjusted estimates and their precision (eg, 95% confidence interval). Make clear which confounders were adjusted for and why they were included	NA
		(*b*) Report category boundaries when continuous variables were categorized	NA
		(*c*) If relevant, consider translating estimates of relative risk into absolute risk for a meaningful time period	NA
Other analyses	17	Report other analyses done—eg analyses of subgroups and interactions, and sensitivity analyses	NA
**Discussion**			
Key results	18	Summarise key results with reference to study objectives	9-12
Limitations	19	Discuss limitations of the study, taking into account sources of potential bias or imprecision. Discuss both direction and magnitude of any potential bias	12-13
Interpretation	20	Give a cautious overall interpretation of results considering objectives, limitations, multiplicity of analyses, results from similar studies, and other relevant evidence	9-12
Generalisability	21	Discuss the generalisability (external validity) of the study results	12
**Other information**			
Funding	22	Give the source of funding and the role of the funders for the present study and, if applicable, for the original study on which the present article is based	13

*Give information separately for exposed and unexposed groups.

Note: An Explanation and Elaboration article discusses each checklist item and gives methodological background and published examples of transparent reporting. The STROBE checklist is best used in conjunction with this article (freely available on the Web sites of PLoS Medicine at http://www.plosmedicine.org/, Annals of Internal Medicine at http://www.annals.org/, and Epidemiology at http://www.epidem.com/). Information on the STROBE Initiative is available at http://www.strobe-statement.org.

For peer review only

For peer review only - http://bmjopen.bmj.com/site/about/guidelines.xhtml
